# Crystal structure and Hirshfeld surfaces analysis of the nickel(II) complex of the Shiff base ligand 6,6′-{(1*E*,1′*E*)-[ethane-1,2-diylbis(aza­nylyl­idene)]bis­(methanylyl­idene)}bis­[2-(tri­fluoro­meth­oxy)phenol]

**DOI:** 10.1107/S2056989019001919

**Published:** 2019-02-08

**Authors:** Sibel Demir Kanmazalp, Seher Meral, Necmi Dege, Aysen Alaman Agar, Igor O. Fritsky

**Affiliations:** a Gaziantep University, Technical Sciences, 27310, Gaziantep, Turkey; b Ondokuz Mayıs University, Faculty of Arts and Sciences, Department of Chemistry, 55139, Kurupelit, Samsun, Turkey; c Ondokuz Mayıs University, Faculty of Arts and Sciences, Department of Physics, 55139, Kurupelit, Samsun, Turkey; d Department of Chemistry, Taras Shevchenko National University of Kyiv, Volodymyrska 64/13, 01601 Kyiv, Ukraine

**Keywords:** crystal structure, nickel(II), square-planar, Schiff base, Ni⋯Ni inter­action, Hirshfeld surfaces analysis

## Abstract

In the title complex, (6,6′-{(1*E*,1′*E*)-[ethane-1,2-diylbis(aza­nylyl­idene)]bis(methanylyl­idene)}bis­[2-(tri­fluoro­meth­oxy)phenol]-κ^4^ *O*,*N*,*N*′,*O*′)nickel(II), [Ni(C_18_H_12_F_6_N_2_O_4_)], the nickel(II) ion has a square-planar coordination sphere, being ligated by two N and two O atoms of the Schiff base ligand 6,6′-{(1*E*,1′*E*)-[ethane-1,2-diylbis(aza­nylyl­idene)]bis­(methanylyl­idene)}bis­[2-(tri­fluoro­meth­oxy)phenol] (**L**). Inversion-related mol­ecules are linked by a short Ni⋯Ni inter­action of 3.2945 (6) Å forming a dimer. In the crystal, the dimers stack up the *a* axis, with a closest Ni⋯Ni separation of *ca* 3.791 Å. There are no other significant inter­molecular inter­actions present. However, the Hirshfeld surface analysis and the two-dimensional fingerprint plots indicate that the packing is dominated by H⋯F/F⋯H, H⋯H, O⋯H/H⋯O and C⋯H/H⋯C contacts.

## Abstract 2

In the title complex, the nickel(II) ion has a square-planar coordination sphere, being ligated by two N and two O atoms of the tetra­dentate Schiff base ligand 6,6′-{(1*E*,1′*E*)-[ethane-1,2-diylbis(aza­nylyl­idene]bis­(methanylyl­idene)}bis­[2-(tri­fluoro­meth­oxy)phenol].

## Chemical context   

Schiff bases complexes with metals are the focus of many areas of research such as the inter­action of biomolecules with metals and the biological effects of metal complexes. Their —OH and C=N groups are involved in the formation of covalent bonding with the metal atom; besides, these mol­ecules are known to be easy to synthesize giving a high yield under mild conditions by solvent or solvent-free methods (Tiwari *et al.*, 2011 ▸; Kumar *et al.*, 2009 ▸; Kundu *et al.*, 2009 ▸). 2-Hy­droxy­benzaldehyde has been used to synthesize salen-type Schiff bases, which consist of an ONNO tetra­dentate ligand and form five- and six-membered chelate rings with a metal atom (Atkins *et al.*, 1985 ▸; Gupta & Sutar, 2008 ▸). The redox character of the metal atom as well as its thermodynamic and kinetic properties results in an increase in the activity of salen-type compounds compared to organic compounds (Rijt & Sadler, 2009 ▸). Nickel is encountered in nature as a toxic metal and therefore synthesizing compounds to selectively remove toxic materials is an important subject of research (Gupta *et al.*, 2008 ▸). In this study, the title nickel(II) complex was synthesized from the salen-type Schiff base, 6,6′-{(1*E*,1′*E*)-[ethane-1,2-diylbis(aza­nylyl­idene)]bis­(methanylyl­idene)}bis­[2-(tri­fluoro­meth­oxy)phenol] (**L**), using nickel acetate and we report herein its crystal structure and the analysis of the Hirshfeld surface.
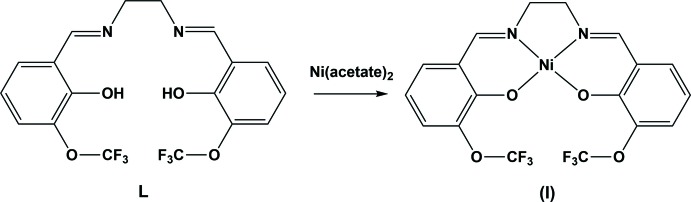


## Structural commentary   

**Figure 1:**
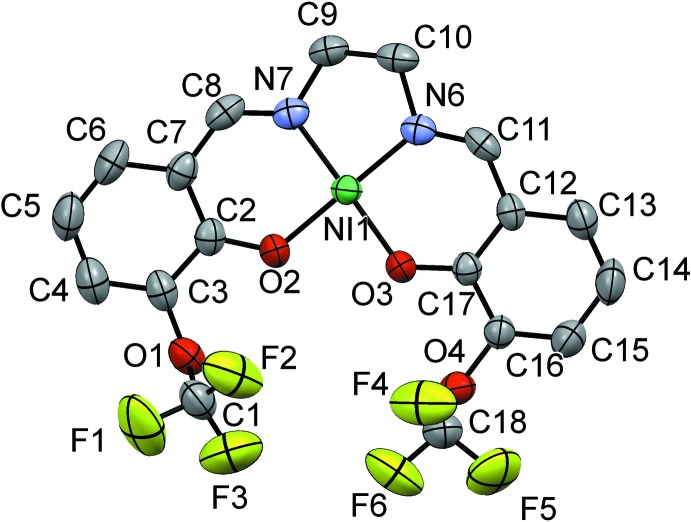
The mol­ecular structure of the asymmetric unit of the title compound with the atom labelling. Displacement ellipsoids are drawn at the 50% probability level.

**Figure 2:**
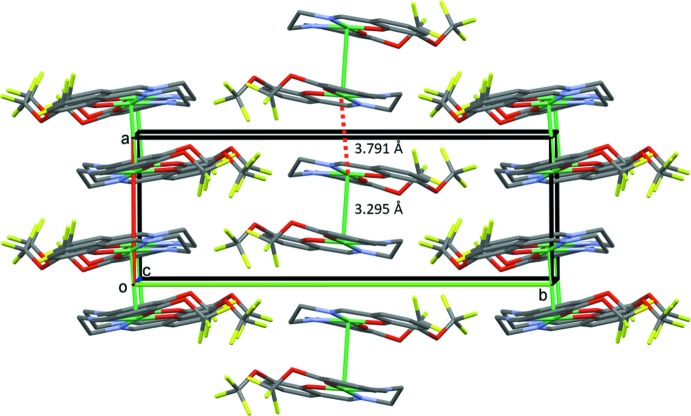
A view along the *c* axis of the crystal packing of the title compound. The various Ni⋯Ni inter­actions are shown as green lines and dashed red lines. H atoms have been omitted for clarity.

The mol­ecular structure of the asymmetric unit of the title compound (I) is shown in Fig. 1 ▸. Inversion-related complex mol­ecules are linked by an Ni1⋯Ni1^i^ inter­metallic *d* ^8^⋯*d* ^8^ inter­action of 3.2945 (6) Å [Fig. 2 ▸; symmetry code (i): −*x* + 1, −*y* + 1, −*z* + 1]. The nickel ion Ni1 is coordinated by two imine N atoms, N6 and N7, and by two phenoxo O atoms, O2 and O3, of the tetra­dentate Schiff base ligand **L**. The bond lengths, Ni—O2 and Ni—O3 [1.845 (2) and 1.840 (2) Å, respectively], and Ni—N6 and Ni—N7 [1.839 (3) and 1.843 (3) Å, respectively] are close to the values observed for nickel complexes of similar ligands (see section *Database survey*). The coordinating atoms, N6, N7, O2, O3, are essentially planar with no atom deviating from its mean plane by more than 0.0325 Å. The τ_4_ factor for four-coordinated metal atoms is = 0.04, indicating an almost perfect square-planar coordination sphere for atom Ni1 (τ_4_ = 0 for a perfect square-planar geometry, = 1 for a perfect tetra­hedral geometry; Yang *et al.*, 2007 ▸).

## Supra­molecular features   

In the crystal, the dimers stack up the *a*-axis direction with a Ni1^i^⋯Ni1^ii^ separation of *ca*. 3.791 Å [see Fig. 2 ▸; symmetry codes: (i): −*x* + 1, −*y* + 1, −*z* + 1; (ii) *x* + 1, *y*, *z*]. There are no other significant inter­molecular inter­actions present; both C—H⋯F and C—H⋯O inter­actions exceed the sum of their van der Walls radii.

## Database survey   

A search of the Cambridge Structural Database (CSD, Version 5.40, November 2018; Groom *et al.*, 2016 ▸) for a 2,2′-[ethane-1,2-diylbis(imino­methyl­idene)]bis­(phenolato)]nickel(II) moiety but with different substituents on the aromatic rings gave over 60 hits. Apart from the search skeleton (CSD refcode SAENNI), whose structure was first reported by Shkol’nikova *et al.* (1970 ▸), the majority of the compounds involve bis­(6-meth­oxy­phenolato) and bis­(6-eth­oxy­phenalato) groups [see supporting information files S1(H), S2(OMe) and S3(OEt)]. A common feature of these complexes is the dimer formation with an Ni⋯Ni separation of between *ca* 3.2 to 3.9 Å. The same dimeric arrangement is found in the title complex, where this separation is 3.2945 (6) Å. In the majority of these complexes, the Ni—N_imine_ bond lengths vary from *ca* 1.837 to 1.956 Å while the Ni—O_phenoxo_ bond lengths vary from *ca* 1.834 to 1.936 Å. In the title complex, the Ni—N_imine_ [1.839 (3) and 1.843 (3) Å] and Ni—O_phenoxo_ [1.840 (2) and 1.845 (2) Å] bond lengths fall within these limits.

## Hirshfeld surface analysis   

**Figure 3:**
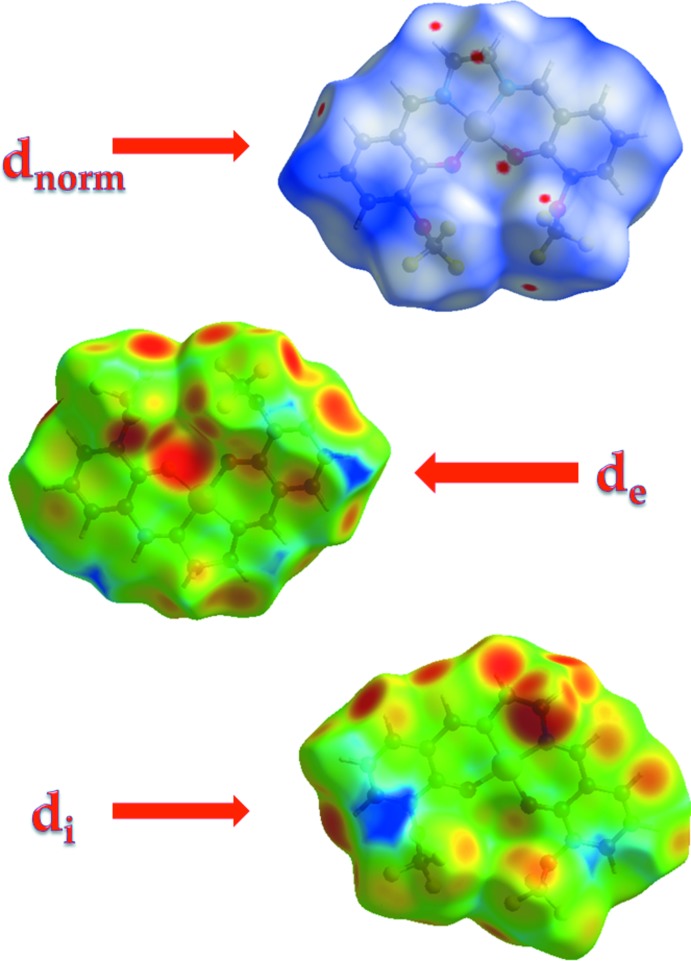
The Hirshfeld surface mapped over *d*_norm_, *d*_i_ and *d*_e_.

**Figure 5:**
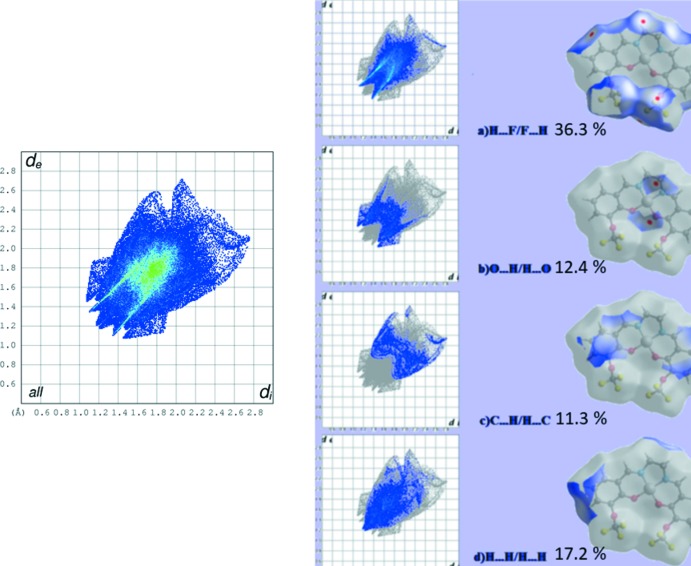
Total two-dimensional fingerprint plot (left) and the individual contributions to the Hirshfeld surface, together with areas of Hirshfeld surfaces involved in the inter­molecular contacts (right).

**Figure 6:**
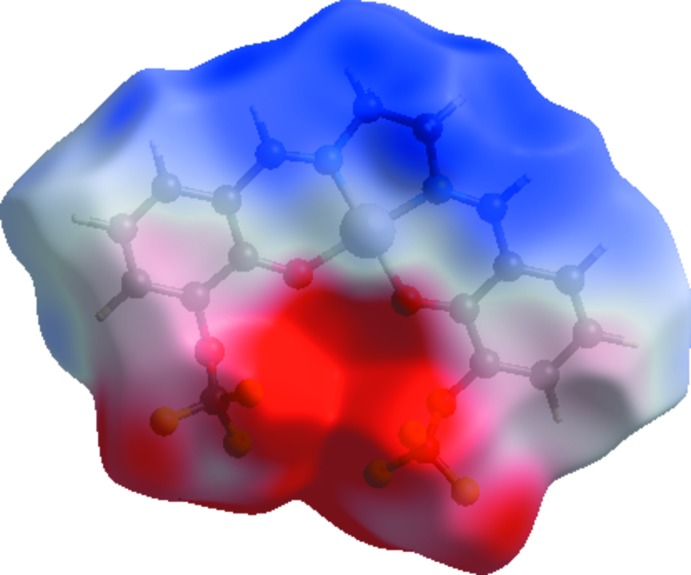
Electrostatic potential surface for the title compound.

The Hirshfeld surface analysis (Spackman & Jayatilaka, 2009 ▸) and the associated two-dimensional fingerprint plots (McKinnon *et al.*, 2007 ▸) were performed with *CrystalExplorer17* (Turner *et al.*, 2017 ▸). Hirshfeld surfaces enable the visualization of inter­molecular inter­actions by using different colours and colour intensity to represent short or long contacts and indicate of the relative strength of the inter­actions. The red regions indicate areas of close contacts shorter than the sum of van der Waals radii, while the blue and white regions represent contacts having distances greater and equal to the sum of van der Waals radii, respectively. The three-dimensional Hirshfeld surfaces calculated for the title compound are depicted in Figs. 3 ▸ and 4 ▸. A qu­anti­tative estimate of the inter­molecular inter­actions in the crystal structure of the title compound was obtained using Hirshfeld analysis with 2D fingerprint plots (Fig. 5 ▸). As can be seen from the individual fingerprint plots (Fig. 5 ▸), the most dominant contribution to the Hirshfeld surface is from F⋯H/H⋯F inter­actions, with a value equal to 36.3%. The scattering points spread up to *d*_e_ = *d*_i_ = 1.4 Å. The other dominant forces are H⋯H (17.2%), O⋯H (12.4%) and C⋯H (11.3%) contacts. The electrostatic potential energy in the range −0.031 to 0.256 a.u., obtained using the STO-3G basis set at the Hartree–Fock level of theory, is illustrated in Fig. 6 ▸. The C—H⋯O and C—H⋯F donors and acceptors are shown as blue and red areas around the atoms with positive (donor) and negative (acceptors) electrostatic potentials.

## Synthesis and crystallization   

The title Schiff base ligand (**L**), was synthesized by condensation of 2-hy­droxy-3-tri­fluoro­meth­oxy­benzaldehyde (0.0095 mmol) and 1,2-ethanedi­amine (0.0095 mmol) in ethanol under reflux for *ca* 18 h. The yellow product obtained was washed with ether and dried at room temperature. Ni(CH_3_COO)_2_·4H_2_O (0.0080 mmol) dissolved in 20 ml of ethanol was added slowly to an ethanol (20 ml) solution of **L** (0.0080 mmol) and the mixture was refluxed for *ca* 6 h. The orange product obtained was filtered off and washed with toluene. Red rod-like crystals of the title complex were obtained by slow evaporation of a solution in ethanol at room temperature (yield 82%, m.p. > 673 K).

## Refinement   

Crystal data, data collection and structure refinement details are summarized in Table 1 ▸. All H atoms were positioned with idealized geometry and refined as riding: C—H = 0.93–0.97 Å with *U*_iso_(H) = 1.2*U*_eq_(C).

## Supplementary Material

Crystal structure: contains datablock(s) I, Global. DOI: 10.1107/S2056989019001919/su5478sup1.cif

Structure factors: contains datablock(s) I. DOI: 10.1107/S2056989019001919/su5478Isup2.hkl

CSD search S1. DOI: 10.1107/S2056989019001919/su5478sup3.pdf

CSD search S2. DOI: 10.1107/S2056989019001919/su5478sup4.pdf

CSD search S3. DOI: 10.1107/S2056989019001919/su5478sup5.pdf

CCDC reference: 1890705

Additional supporting information:  crystallographic information; 3D view; checkCIF report

## Figures and Tables

**Figure 4:**
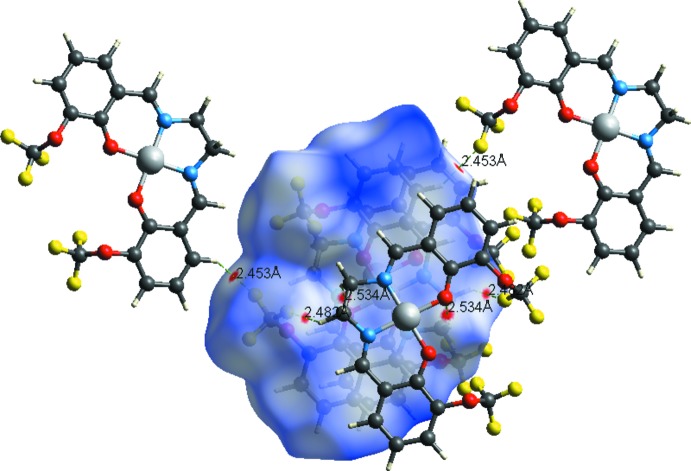
Hirshfeld surface mapped over *d*_norm_, showing the weak inter­molecular C—H⋯O and C—H⋯F contacts.

**Table 1:** Experimental details

Crystal data	
Chemical formula	[Ni(C_18_H_12_F_6_N_2_O_4_)]
*M*_r_	493.01
Crystal system, space group	Monoclinic, *P*2_1_/*n*
Temperature (K)	296
*a*, *b*, *c* (Å)	7.0709 (4), 19.8158 (13), 13.1957 (7)
β (°)	99.089 (4)
*V* (Å^3^)	1825.71 (19)
*Z*	4
Radiation type	Mo *K*α
μ (mm^−1^)	1.15
Crystal size (mm)	0.43 × 0.19 × 0.05

Data collection	
Diffractometer	Stoe *IPDS* 2
Absorption correction	Integration (*X-RED32*; Stoe & Cie, 2002 ▸)
*T*_min_, *T*_max_	0.752, 0.954
No. of measured, independent and observed [*I* > 2σ(*I*)] reflections	10231, 3594, 2004
*R*_int_	0.069
(sin θ/λ)_max_ (Å^−1^)	0.617

Refinement	
*R*[*F* ^2^ > 2σ(*F* ^2^)], *wR*(*F* ^2^), *S*	0.042, 0.073, 0.82
No. of reflections	3594
No. of parameters	280
H-atom treatment	H-atom parameters constrained
Δρ_max_, Δρ_min_ (e Å^−3^)	0.28, −0.26

Computer programs: *X-AREA* and *X-RED32* (Stoe & Cie, 2002 ▸), *SHELXT2018* (Sheldrick, 2015*a* ▸), *SHELXL2018* (Sheldrick, 2015*b* ▸), *Mercury* (Macrae *et al.*, 2008 ▸), *ORTEP-3 for Windows* and *WinGX* (Farrugia, 2012 ▸) and *PLATON* (Spek, 2009 ▸).

## supplementary crystallographic information

### Crystal data

**Table:**

[Ni(C_18_H_12_F_6_N_2_O_4_)]	*F*(000) = 992
*M**_r_* = 493.01	*D*_x_ = 1.794 Mg m^−^^3^
Monoclinic, *P*2_1_/*n*	Mo *K*α radiation, λ = 0.71073 Å
*a* = 7.0709 (4) Å	Cell parameters from 7677 reflections
*b* = 19.8158 (13) Å	θ = 1.9–29.8°
*c* = 13.1957 (7) Å	µ = 1.15 mm^−^^1^
β = 99.089 (4)°	*T* = 296 K
*V* = 1825.71 (19) Å^3^	Rod, red
*Z* = 4	0.43 × 0.19 × 0.05 mm

### Data collection

**Table:**

Stoe IPDS 2 diffractometer	3594 independent reflections
Radiation source: sealed X-ray tube, 12 x 0.4 mm long-fine focus	2004 reflections with *I* > 2σ(*I*)
Detector resolution: 6.67 pixels mm^-1^	*R*_int_ = 0.069
rotation method scans	θ_max_ = 26.0°, θ_min_ = 1.9°
Absorption correction: integration (X-RED32; Stoe & Cie, 2002)	*h* = −8→8
*T*_min_ = 0.752, *T*_max_ = 0.954	*k* = −22→24
10231 measured reflections	*l* = −16→16

### Refinement

**Table:**

Refinement on *F*^2^	Primary atom site location: structure-invariant direct methods
Least-squares matrix: full	Secondary atom site location: difference Fourier map
*R*[*F*^2^ > 2σ(*F*^2^)] = 0.042	Hydrogen site location: inferred from neighbouring sites
*wR*(*F*^2^) = 0.073	H-atom parameters constrained
*S* = 0.82	*w* = 1/[σ^2^(*F*_o_^2^) + (0.0208*P*)^2^] where *P* = (*F*_o_^2^ + 2*F*_c_^2^)/3
3594 reflections	(Δ/σ)_max_ < 0.001
280 parameters	Δρ_max_ = 0.28 e Å^−^^3^
0 restraints	Δρ_min_ = −0.26 e Å^−^^3^

### Special details

**Table:**

Geometry. All esds (except the esd in the dihedral angle between two l.s. planes) are estimated using the full covariance matrix. The cell esds are taken into account individually in the estimation of esds in distances, angles and torsion angles; correlations between esds in cell parameters are only used when they are defined by crystal symmetry. An approximate (isotropic) treatment of cell esds is used for estimating esds involving l.s. planes.
Refinement. Refinement of F^2^ against ALL reflections. The weighted R-factor wR and goodness of fit S are based on F^2^, conventional R-factors R are based on F, with F set to zero for negative F^2^. The threshold expression of F^2^ > 2sigma(F^2^) is used only for calculating R-factors(gt) etc. and is not relevant to the choice of reflections for refinement. R-factors based on F^2^ are statistically about twice as large as those based on F, and R- factors based on ALL data will be even larger.

### Fractional atomic coordinates and isotropic or equivalent isotropic displacement parameters (Å^2^)

**Table:**

	*x*	*y*	*z*	*U*_iso_*/*U*_eq_	
Ni1	0.26730 (6)	0.49434 (2)	0.49904 (4)	0.03711 (13)	
O3	0.2821 (4)	0.40837 (12)	0.55236 (18)	0.0425 (6)	
O2	0.3199 (3)	0.45369 (12)	0.38085 (18)	0.0449 (7)	
O4	0.3829 (4)	0.28127 (12)	0.60619 (19)	0.0507 (7)	
O1	0.4119 (4)	0.37488 (14)	0.22988 (19)	0.0566 (7)	
N6	0.2039 (4)	0.53396 (14)	0.6151 (2)	0.0411 (8)	
N7	0.2627 (4)	0.58028 (15)	0.4452 (3)	0.0438 (8)	
F2	0.1222 (5)	0.33925 (16)	0.2439 (3)	0.1148 (12)	
F4	0.1024 (4)	0.26758 (15)	0.5082 (2)	0.1128 (12)	
F6	0.3485 (5)	0.21399 (17)	0.4791 (2)	0.1136 (11)	
F5	0.2146 (5)	0.18837 (15)	0.6022 (3)	0.1122 (11)	
C17	0.2694 (5)	0.39078 (18)	0.6458 (3)	0.0381 (9)	
C2	0.3145 (5)	0.4817 (2)	0.2913 (3)	0.0434 (10)	
F1	0.2352 (5)	0.31439 (18)	0.1122 (2)	0.1288 (14)	
C12	0.2217 (5)	0.4342 (2)	0.7222 (3)	0.0413 (9)	
C11	0.1888 (4)	0.5050 (2)	0.7009 (3)	0.0443 (10)	
H11	0.154060	0.531744	0.752904	0.053*	
F3	0.3513 (6)	0.27078 (17)	0.2496 (3)	0.1309 (13)	
C3	0.3510 (6)	0.4423 (2)	0.2078 (3)	0.0501 (10)	
C7	0.2774 (5)	0.5505 (2)	0.2689 (3)	0.0470 (10)	
C16	0.3104 (5)	0.32354 (19)	0.6763 (3)	0.0437 (9)	
C8	0.2613 (5)	0.5966 (2)	0.3501 (3)	0.0496 (11)	
H8	0.248688	0.642164	0.333355	0.060*	
C10	0.1626 (6)	0.60683 (18)	0.6044 (3)	0.0524 (11)	
H10A	0.201695	0.629815	0.669248	0.063*	
H10B	0.026750	0.614357	0.582588	0.063*	
C9	0.2752 (6)	0.63223 (18)	0.5249 (3)	0.0508 (11)	
H9A	0.221970	0.674426	0.495980	0.061*	
H9B	0.407695	0.639754	0.555126	0.061*	
C13	0.2102 (6)	0.4102 (2)	0.8210 (3)	0.0566 (11)	
H13	0.175958	0.439462	0.870112	0.068*	
C1	0.2811 (8)	0.3271 (3)	0.2096 (4)	0.0674 (13)	
C18	0.2617 (7)	0.2405 (2)	0.5502 (4)	0.0616 (12)	
C15	0.3026 (6)	0.3011 (2)	0.7727 (3)	0.0619 (12)	
H15	0.333337	0.256546	0.790046	0.074*	
C6	0.2706 (6)	0.5749 (2)	0.1687 (4)	0.0646 (13)	
H6	0.245387	0.620387	0.155453	0.078*	
C14	0.2486 (7)	0.3447 (3)	0.8456 (3)	0.0709 (14)	
H14	0.238953	0.328986	0.910957	0.085*	
C4	0.3449 (7)	0.4673 (3)	0.1098 (3)	0.0675 (13)	
H4	0.371002	0.439315	0.057199	0.081*	
C5	0.3000 (7)	0.5337 (3)	0.0908 (4)	0.0748 (15)	
H5	0.289649	0.550545	0.024387	0.090*	

### Atomic displacement parameters (Å^2^)

**Table:**

	*U*^11^	*U*^22^	*U*^33^	*U*^12^	*U*^13^	*U*^23^
Ni1	0.0390 (2)	0.0343 (2)	0.0376 (2)	0.0013 (3)	0.00489 (16)	−0.0016 (3)
O3	0.0528 (16)	0.0404 (14)	0.0359 (15)	0.0000 (12)	0.0115 (12)	−0.0022 (11)
O2	0.0556 (16)	0.0446 (15)	0.0351 (15)	−0.0001 (12)	0.0089 (13)	−0.0010 (12)
O4	0.0550 (17)	0.0421 (15)	0.0554 (17)	0.0010 (13)	0.0096 (14)	−0.0003 (13)
O1	0.0583 (18)	0.0611 (19)	0.0513 (16)	−0.0040 (15)	0.0117 (14)	−0.0148 (14)
N6	0.0360 (17)	0.0402 (17)	0.0457 (19)	0.0028 (14)	0.0020 (15)	−0.0035 (15)
N7	0.0380 (18)	0.0406 (19)	0.051 (2)	0.0021 (14)	0.0015 (15)	0.0006 (16)
F2	0.094 (2)	0.101 (2)	0.168 (3)	−0.0372 (19)	0.077 (2)	−0.050 (2)
F4	0.090 (2)	0.088 (2)	0.140 (3)	0.0169 (17)	−0.047 (2)	−0.0251 (19)
F6	0.119 (3)	0.116 (3)	0.108 (2)	0.001 (2)	0.023 (2)	−0.058 (2)
F5	0.134 (3)	0.068 (2)	0.133 (3)	−0.0416 (19)	0.017 (2)	0.0097 (19)
C17	0.036 (2)	0.043 (2)	0.035 (2)	−0.0044 (17)	0.0062 (17)	0.0002 (17)
C2	0.037 (2)	0.053 (3)	0.040 (2)	−0.0071 (18)	0.0055 (17)	0.0028 (19)
F1	0.153 (3)	0.159 (3)	0.077 (2)	−0.078 (3)	0.025 (2)	−0.052 (2)
C12	0.038 (2)	0.054 (3)	0.032 (2)	−0.0006 (19)	0.0047 (17)	0.0009 (18)
C11	0.0370 (19)	0.054 (3)	0.042 (2)	0.003 (2)	0.0070 (16)	−0.017 (2)
F3	0.160 (3)	0.065 (2)	0.167 (3)	−0.002 (2)	0.023 (3)	0.004 (2)
C3	0.046 (2)	0.068 (3)	0.037 (2)	−0.007 (2)	0.0087 (19)	−0.005 (2)
C7	0.041 (2)	0.052 (3)	0.046 (2)	−0.009 (2)	0.003 (2)	0.010 (2)
C16	0.048 (2)	0.044 (2)	0.039 (2)	−0.0041 (18)	0.0069 (18)	0.0016 (18)
C8	0.039 (2)	0.041 (2)	0.067 (3)	−0.0015 (19)	0.004 (2)	0.015 (2)
C10	0.052 (3)	0.038 (2)	0.065 (3)	0.0063 (18)	0.002 (2)	−0.012 (2)
C9	0.054 (3)	0.035 (2)	0.062 (3)	−0.0007 (19)	0.006 (2)	−0.0013 (18)
C13	0.063 (3)	0.069 (3)	0.039 (2)	−0.008 (2)	0.012 (2)	−0.005 (2)
C1	0.081 (4)	0.070 (4)	0.055 (3)	−0.011 (3)	0.022 (3)	−0.015 (3)
C18	0.074 (3)	0.049 (3)	0.062 (3)	−0.001 (3)	0.012 (3)	−0.002 (2)
C15	0.079 (3)	0.052 (3)	0.055 (3)	−0.004 (2)	0.010 (2)	0.010 (2)
C6	0.068 (3)	0.066 (3)	0.059 (3)	−0.001 (2)	0.006 (2)	0.029 (2)
C14	0.091 (4)	0.081 (4)	0.042 (3)	−0.003 (3)	0.015 (2)	0.017 (3)
C4	0.070 (3)	0.091 (4)	0.044 (3)	−0.008 (3)	0.016 (2)	−0.002 (2)
C5	0.092 (4)	0.088 (4)	0.045 (3)	−0.008 (3)	0.012 (3)	0.018 (3)

### Geometric parameters (Å, º)

**Table:**

Ni1—N6	1.839 (3)	C12—C11	1.443 (5)
Ni1—O3	1.840 (2)	C11—H11	0.9300
Ni1—N7	1.843 (3)	F3—C1	1.299 (5)
Ni1—O2	1.845 (2)	C3—C4	1.379 (5)
O3—C17	1.297 (4)	C7—C6	1.401 (5)
O2—C2	1.301 (4)	C7—C8	1.427 (6)
O4—C18	1.316 (5)	C16—C15	1.357 (5)
O4—C16	1.403 (4)	C8—H8	0.9300
O1—C1	1.321 (5)	C10—C9	1.501 (6)
O1—C3	1.419 (5)	C10—H10A	0.9700
N6—C11	1.288 (5)	C10—H10B	0.9700
N6—C10	1.475 (4)	C9—H9A	0.9700
N7—C8	1.295 (5)	C9—H9B	0.9700
N7—C9	1.464 (5)	C13—C14	1.354 (6)
F2—C1	1.298 (5)	C13—H13	0.9300
F4—C18	1.291 (5)	C15—C14	1.390 (6)
F6—C18	1.311 (5)	C15—H15	0.9300
F5—C18	1.313 (5)	C6—C5	1.355 (6)
C17—C12	1.407 (5)	C6—H6	0.9300
C17—C16	1.409 (5)	C14—H14	0.9300
C2—C3	1.409 (5)	C4—C5	1.367 (6)
C2—C7	1.410 (5)	C4—H4	0.9300
F1—C1	1.300 (5)	C5—H5	0.9300
C12—C13	1.402 (5)		
			
N6—Ni1—O3	94.78 (12)	N6—C10—H10A	110.5
N6—Ni1—N7	86.27 (14)	C9—C10—H10A	110.5
O3—Ni1—N7	177.70 (14)	N6—C10—H10B	110.5
N6—Ni1—O2	177.53 (13)	C9—C10—H10B	110.5
O3—Ni1—O2	84.95 (11)	H10A—C10—H10B	108.7
N7—Ni1—O2	94.08 (14)	N7—C9—C10	106.7 (3)
C17—O3—Ni1	127.1 (2)	N7—C9—H9A	110.4
C2—O2—Ni1	126.9 (2)	C10—C9—H9A	110.4
C18—O4—C16	117.5 (3)	N7—C9—H9B	110.4
C1—O1—C3	116.9 (3)	C10—C9—H9B	110.4
C11—N6—C10	118.5 (4)	H9A—C9—H9B	108.6
C11—N6—Ni1	127.4 (3)	C14—C13—C12	120.6 (4)
C10—N6—Ni1	114.1 (3)	C14—C13—H13	119.7
C8—N7—C9	120.7 (3)	C12—C13—H13	119.7
C8—N7—Ni1	127.0 (3)	F2—C1—F3	108.3 (5)
C9—N7—Ni1	112.2 (2)	F2—C1—F1	106.6 (4)
O3—C17—C12	125.3 (3)	F3—C1—F1	104.7 (4)
O3—C17—C16	118.9 (3)	F2—C1—O1	114.4 (4)
C12—C17—C16	115.8 (3)	F3—C1—O1	108.8 (4)
O2—C2—C3	119.4 (4)	F1—C1—O1	113.4 (4)
O2—C2—C7	125.6 (4)	F4—C18—F6	109.6 (4)
C3—C2—C7	115.0 (4)	F4—C18—F5	106.0 (4)
C13—C12—C17	121.0 (4)	F6—C18—F5	104.2 (4)
C13—C12—C11	118.7 (4)	F4—C18—O4	115.7 (4)
C17—C12—C11	120.3 (3)	F6—C18—O4	108.1 (4)
N6—C11—C12	124.8 (4)	F5—C18—O4	112.7 (4)
N6—C11—H11	117.6	C16—C15—C14	120.1 (4)
C12—C11—H11	117.6	C16—C15—H15	119.9
C4—C3—C2	123.5 (4)	C14—C15—H15	119.9
C4—C3—O1	119.7 (4)	C5—C6—C7	121.4 (4)
C2—C3—O1	116.7 (3)	C5—C6—H6	119.3
C6—C7—C2	120.6 (4)	C7—C6—H6	119.3
C6—C7—C8	119.5 (4)	C13—C14—C15	119.7 (4)
C2—C7—C8	119.7 (4)	C13—C14—H14	120.1
C15—C16—O4	119.7 (4)	C15—C14—H14	120.1
C15—C16—C17	122.7 (4)	C5—C4—C3	119.4 (5)
O4—C16—C17	117.2 (3)	C5—C4—H4	120.3
N7—C8—C7	125.4 (4)	C3—C4—H4	120.3
N7—C8—H8	117.3	C6—C5—C4	120.0 (5)
C7—C8—H8	117.3	C6—C5—H5	120.0
N6—C10—C9	106.1 (3)	C4—C5—H5	120.0
			
N6—Ni1—O3—C17	−6.3 (3)	C18—O4—C16—C15	88.3 (4)
O2—Ni1—O3—C17	176.1 (3)	C18—O4—C16—C17	−98.9 (4)
O3—Ni1—O2—C2	171.3 (3)	O3—C17—C16—C15	179.3 (4)
N7—Ni1—O2—C2	−10.8 (3)	C12—C17—C16—C15	0.8 (6)
O3—Ni1—N6—C11	2.6 (3)	O3—C17—C16—O4	6.7 (5)
N7—Ni1—N6—C11	−175.3 (3)	C12—C17—C16—O4	−171.8 (3)
O3—Ni1—N6—C10	−175.9 (2)	C9—N7—C8—C7	170.4 (3)
N7—Ni1—N6—C10	6.2 (2)	Ni1—N7—C8—C7	−5.0 (6)
N6—Ni1—N7—C8	−166.3 (3)	C6—C7—C8—N7	179.0 (4)
O2—Ni1—N7—C8	11.3 (3)	C2—C7—C8—N7	−5.8 (6)
N6—Ni1—N7—C9	18.0 (2)	C11—N6—C10—C9	153.9 (3)
O2—Ni1—N7—C9	−164.5 (2)	Ni1—N6—C10—C9	−27.4 (3)
Ni1—O3—C17—C12	7.0 (5)	C8—N7—C9—C10	147.0 (3)
Ni1—O3—C17—C16	−171.4 (2)	Ni1—N7—C9—C10	−36.9 (3)
Ni1—O2—C2—C3	−177.2 (3)	N6—C10—C9—N7	39.5 (4)
Ni1—O2—C2—C7	3.9 (5)	C17—C12—C13—C14	1.3 (6)
O3—C17—C12—C13	179.5 (3)	C11—C12—C13—C14	−177.0 (4)
C16—C17—C12—C13	−2.1 (5)	C3—O1—C1—F2	−48.2 (6)
O3—C17—C12—C11	−2.2 (6)	C3—O1—C1—F3	−169.5 (4)
C16—C17—C12—C11	176.1 (3)	C3—O1—C1—F1	74.4 (6)
C10—N6—C11—C12	179.1 (3)	C16—O4—C18—F4	46.5 (6)
Ni1—N6—C11—C12	0.6 (5)	C16—O4—C18—F6	169.7 (3)
C13—C12—C11—N6	176.5 (3)	C16—O4—C18—F5	−75.7 (5)
C17—C12—C11—N6	−1.7 (5)	O4—C16—C15—C14	173.7 (4)
O2—C2—C3—C4	178.7 (4)	C17—C16—C15—C14	1.4 (6)
C7—C2—C3—C4	−2.3 (6)	C2—C7—C6—C5	−0.3 (7)
O2—C2—C3—O1	−6.5 (5)	C8—C7—C6—C5	174.9 (4)
C7—C2—C3—O1	172.5 (3)	C12—C13—C14—C15	0.9 (7)
C1—O1—C3—C4	−84.6 (5)	C16—C15—C14—C13	−2.2 (7)
C1—O1—C3—C2	100.4 (4)	C2—C3—C4—C5	−0.5 (7)
O2—C2—C7—C6	−178.5 (4)	O1—C3—C4—C5	−175.1 (4)
C3—C2—C7—C6	2.6 (6)	C7—C6—C5—C4	−2.6 (8)
O2—C2—C7—C8	6.4 (6)	C3—C4—C5—C6	3.0 (8)
C3—C2—C7—C8	−172.5 (3)		

### Hydrogen-bond geometry (Å, º)

**Table:**

*D*—H···*A*	*D*—H	H···*A*	*D*···*A*	*D*—H···*A*
C10—H10*B*···O3^i^	0.97	2.63	3.494 (4)	149
C9—H9*A*···F4^i^	0.97	2.56	3.301 (5)	133
C6—H6···F6^ii^	0.93	2.58	3.405 (5)	148

Symmetry codes: (i) −*x*, −*y*+1, −*z*+1; (ii) −*x*+1/2, *y*+1/2, −*z*+1/2.
