# Supplementary material for: Crystal structure and Hirshfeld surfaces analysis of the nickel(II) complex of the Shiff base ligand 6,6′-{(1E,1′E)-[ethane-1,2-diylbis(aza­nylyl­idene)]bis­(methanylyl­idene)}bis­[2-(tri­fluoro­meth­oxy)phenol]
Source: Acta Crystallogr E Crystallogr Commun. 2019 Feb 8;75(Pt 3):328–31. doi: 10.1107/S2056989019001919 (PMC6399694; doi:10.1107/S2056989019001919)

Search Overview

Search:	search7
Date/Time done:	Wed Jan 30 14:32:42 2019
Database(s):	CSD version 5.40 (November 2018)
Restriction Info:	No refcode restrictions applied
Filters:	None
Percentage Completed:	100%
Number of Hits:	28

Single query used. Search found structures that:

match

Query 1

Query 1

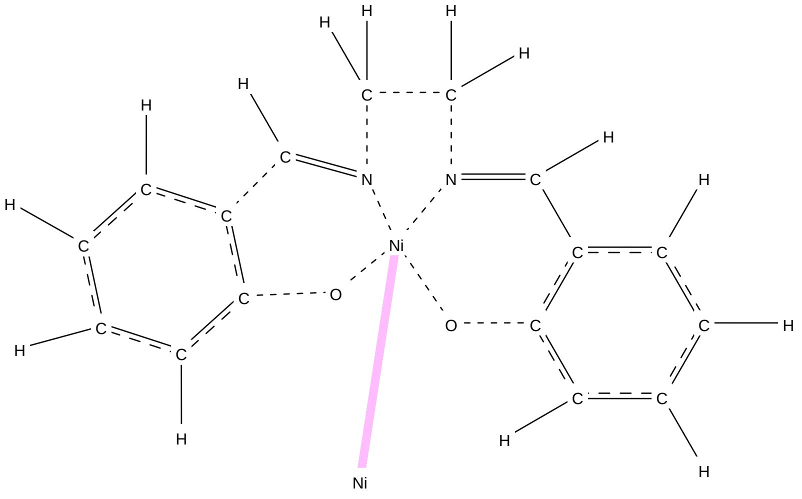

Search: search7 (Wed Jan 30 14:32:42 2019): Hits 1-4

BEXKUK

Reference: A.Giacomelli, C.Floriani, G.Perego (1982) *Chem.Commun.* ,650

Formula: $3(C_{16}\,H_{14}\,N_2\,Ni_1\,O_2),C_1\,H_6\,N_3\,^{1+},C_{24}\,H_{20}\,B_1\,^{1-},2(C_4\,H_8\,O_1)$

Compound Name: tris(N,N'-Ethylene-bis(salicylideneaminato)-nickel(ii)) guanidinium tetraphenylborate tetrahydrofuran solvate

Space Group:	P-1	***Cell:***	***a*** 16.496(8)	***b*** 19.228(8)	***c*** 15.226(4)
Space Group No.:	2	***(Å,°)***	α 94.88(3)	β 102.83(3)	γ 123.81(3)

R-Factor (%): 5.80 ***Temperature(K):*** 295 ***Density(g/cm³):*** 1.316

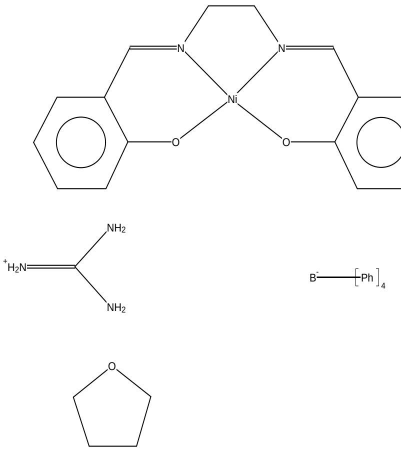

Parameters

Fragment 1	
**DIST1 (D)**	3.338
**DIST2 (D)**	1.839
**DIST3 (D)**	1.880
**DIST4 (D)**	1.835
**DIST5 (D)**	1.847
**ANG1 (A)**	3.223
Fragment 2	
**DIST1 (D)**	3.352
**DIST2 (D)**	1.859
**DIST3 (D)**	1.853
**DIST4 (D)**	1.841
**DIST5 (D)**	1.859
**ANG1 (A)**	6.236
Fragment 3	
**DIST1 (D)**	3.884
**DIST2 (D)**	1.861
**DIST3 (D)**	1.845
**DIST4 (D)**	1.855
**DIST5 (D)**	1.857
**ANG1 (A)**	15.173

COXNIN

Reference: M.A.Siegler, M.Lutz (2009) *Cryst.Growth Des.* ,**9,**1194

Formula: $C_{16}\,H_{14}\,N_2\,Ni_1\,O_2,C_1\,H_2\,Cl_2$

Compound Name: (N,N'-Ethylene-bis(salicylideneiminato))-nickel(ii) dichloromethane solvate

Space Group:	P21/n	***Cell:***	***a*** 13.155(0)	***b*** 6.802(0)	***c*** 19.185(0)
Space Group No.:	14	***(Å,°)***	α 90.00	β 91.00(0)	γ 90.00

R-Factor (%): 3.97 ***Temperature(K):*** 150 ***Density(g/cm³):*** 1.586

Parameters

Fragment 1	
**DIST1 (D)**	3.279
**DIST2 (D)**	1.844
**DIST3 (D)**	1.837
**DIST4 (D)**	1.837
**DIST5 (D)**	1.843
**ANG1 (A)**	8.508
Fragment 2	
**DIST1 (D)**	3.662
**DIST2 (D)**	1.844
**DIST3 (D)**	1.837
**DIST4 (D)**	1.837
**DIST5 (D)**	1.843
**ANG1 (A)**	8.508

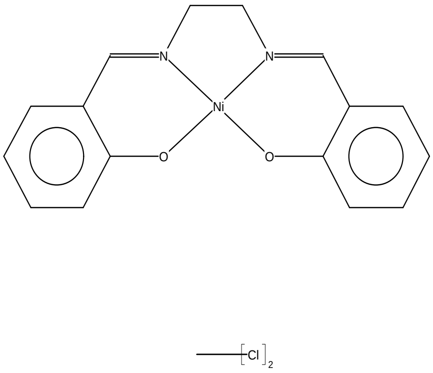

COXNOT

Reference: M.A.Siegler, M.Lutz (2009) *Cryst.Growth Des.* ,**9,**1194

Formula: $C_{16}\,H_{14}\,N_2\,Ni_1\,O_2,C_2\,H_4\,O_2$

Compound Name: (N,N'-Ethylene-bis(salicylideneiminato))-nickel(ii) acetic acid solvate

Space Group:	P-1	***Cell:***	***a*** 11.494(0)	***b*** 7.362(0)	***c*** 10.015(0)
Space Group No.:	2	***(Å,°)***	α 93.81(0)	β 102.23(0)	γ 100.01(0)

R-Factor (%): 4.13 ***Temperature(K):*** 110 ***Density(g/cm³):*** 1.577

Parameters

Fragment 1	
**DIST1 (D)**	3.369
**DIST2 (D)**	1.841
**DIST3 (D)**	1.842
**DIST4 (D)**	1.832
**DIST5 (D)**	1.862
**ANG1 (A)**	10.791

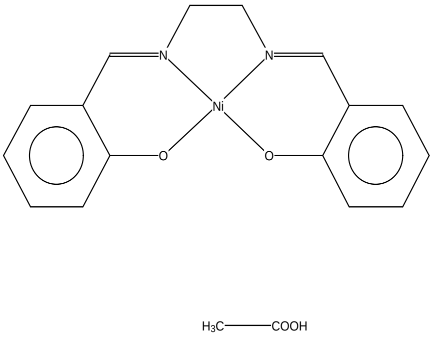

COXNUZ

Reference: M.A.Siegler, M.Lutz (2009) *Cryst.Growth Des.* ,**9,**1194

Formula: $C_{16}\,H_{14}\,N_2\,Ni_1\,O_2,1.5(C_1\,H_4\,O_1)$

Compound Name: (N,N'-Ethylene-bis(salicylideneiminato))-nickel(ii) methanol solvate

Space Group:	Pbcn	***Cell:***	***a*** 18.344(0)	***b*** 6.792(0)	***c*** 26.761(0)
Space Group No.:	60	***(Å,°)***	α 90.00	β 90.00	γ 90.00

R-Factor (%): 2.78 ***Temperature(K):*** 150 ***Density(g/cm³):*** 1.486

Parameters

Fragment 1	
**DIST1 (D)**	3.438
**DIST2 (D)**	1.849
**DIST3 (D)**	1.842
**DIST4 (D)**	1.842
**DIST5 (D)**	1.852
**ANG1 (A)**	9.781

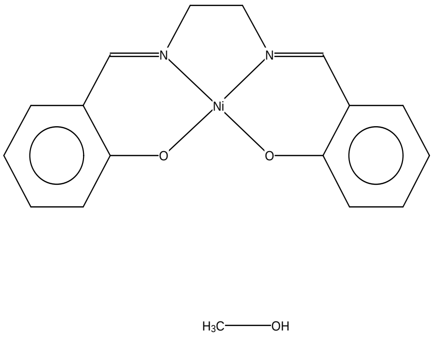

Search: search7 (Wed Jan 30 14:32:42 2019): Hits 5-8

FENRIA

Reference: S.Skovgaard, A.D.Bond, C.J.McKenzie (2005) *Acta Crystallogr.,Sect.E:Struct.Rep.Online* ,**61,**m135

Formula: $2(K_1{}^{1+}),6(C_{16}H_{14}N_2Ni_1O_2),C_4N_4Ni_1{}^{2-},6(C_1H_4O_1),2(H_2O_1)$

Compound Name: Dipotassium hexakis((2,2'-(ethane-1,2-diylbis(nitrilomethylidyne)) diphenolato)-nickel(ii)) tetracyano-nickel(ii) methanol solvate dihydrate

Space Group:	Pbca	***Cell:***	***a***	14.321(1)	***b***	24.855(2)	***c***	29.709(3)
Space Group No.:	61	***(Å,°)***	α	90.00	β	90.00	γ	90.00

R-Factor (%): 5.47 ***Temperature(K):*** 180 ***Density(g/cm³):*** 1.520

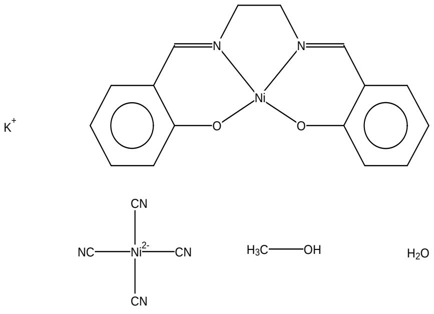

Parameters

Fragment 1	
**DIST1 (D)**	3.322
**DIST2 (D)**	1.833
**DIST3 (D)**	1.846
**DIST4 (D)**	1.859
**DIST5 (D)**	1.850
**ANG1 (A)**	12.926

Fragment 2	
**DIST1 (D)**	3.322
**DIST2 (D)**	1.843
**DIST3 (D)**	1.839
**DIST4 (D)**	1.851
**DIST5 (D)**	1.851
**ANG1 (A)**	1.910

Fragment 3	
**DIST1 (D)**	3.340
**DIST2 (D)**	1.842
**DIST3 (D)**	1.842
**DIST4 (D)**	1.853
**DIST5 (D)**	1.851
**ANG1 (A)**	1.904

FEYWEM

Reference: Xiao-Li Cheng, Shan Gao, Li-Hua Huo, S.W.Ng (2005) *Acta Crystallogr.,Sect.E:Struct.Rep.Online* ,**61,**m385

Formula: $C_{16}H_{14}N_2Ni_1O_2,C_1H_1Cl_3$

Compound Name: (N,N'-Ethylene-bis(salicylideneiminato))-nickel(ii) chloroform solvate

Space Group:	Pnnm	***Cell:***	***a***	6.997(1)	***b***	14.221(3)	***c***	18.355(4)
Space Group No.:	58	***(Å,°)***	α	90.00	β	90.00	γ	90.00

R-Factor (%): 4.60 ***Temperature(K):*** 295 ***Density(g/cm³):*** 1.616

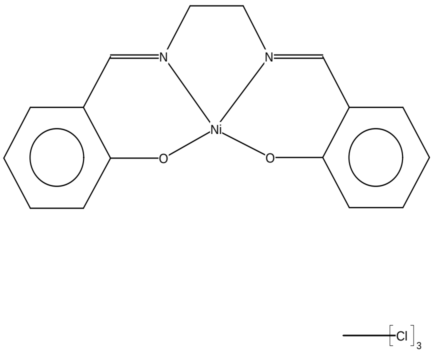

Parameters

Fragment 1	
**DIST1 (D)**	3.191
**DIST2 (D)**	1.843
**DIST3 (D)**	1.843
**DIST4 (D)**	1.844
**DIST5 (D)**	1.844
**ANG1 (A)**	3.843

FEYWEM01

Reference: M.A.Siegler, M.Lutz (2009) *Cryst.Growth Des.* ,**9,**1194

Formula: $C_{16}H_{14}N_2Ni_1O_2,C_1H_1Cl_3$

Compound Name: (N,N'-Ethylene-bis(salicylideneiminato))-nickel(ii) chloroform solvate

Space Group:	P21/n	***Cell:***	***a***	13.863(0)	***b***	6.949(0)	***c***	18.093(0)
Space Group No.:	14	***(Å,°)***	α	90.00	β	90.11(0)	γ	90.00

R-Factor (%): 3.70 ***Temperature(K):*** 110 ***Density(g/cm³):*** 1.694

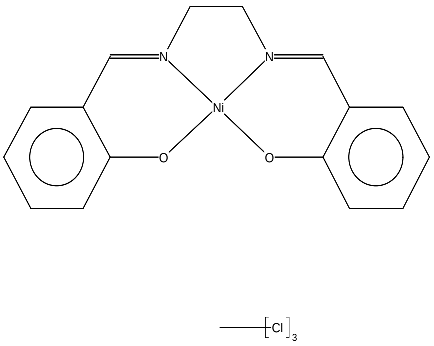

Parameters

Fragment 1	
**DIST1 (D)**	3.133
**DIST2 (D)**	1.850
**DIST3 (D)**	1.835
**DIST4 (D)**	1.839
**DIST5 (D)**	1.857
**ANG1 (A)**	3.759

FEYWEM02

Reference: M.A.Siegler, M.Lutz (2009) *Cryst.Growth Des.* ,**9,**1194

Formula: $C_{16}H_{14}N_2Ni_1O_2,C_1H_1Cl_3$

Compound Name: (N,N'-Ethylene-bis(salicylideneiminato))-nickel(ii) chloroform solvate

Space Group:	Pnnm	***Cell:***	***a***	14.227(0)	***b***	6.995(0)	***c***	18.335(0)
Space Group No.:	58	***(Å,°)***	α	90.00	β	90.00	γ	90.00

R-Factor (%): 4.66 ***Temperature(K):*** 295 ***Density(g/cm³):*** 1.618

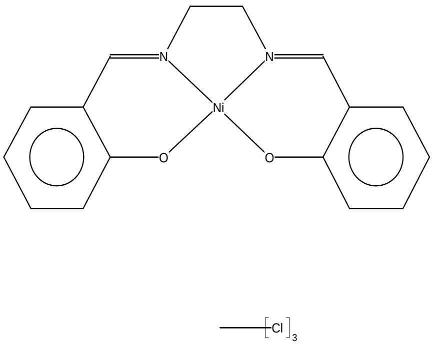

Parameters

Fragment 1	
**DIST1 (D)**	3.193
**DIST2 (D)**	1.840
**DIST3 (D)**	1.840
**DIST4 (D)**	1.847
**DIST5 (D)**	1.847
**ANG1 (A)**	3.620

Search: search7 (Wed Jan 30 14:32:42 2019): Hits 9-12

JIMNOJ

Reference: Xun Feng, Zhong-Xiang Du, Bao-Kian Ye, Fu-Na Cui (2007) *Jiegou Huaxue(Chin.)(Chin.J.Struct.Chem.)* ,**26,**1033

Formula: 2(C_{16} H_{14} N_2 Ni_1 O_2),H_4 N_1 $^{1+}$,C_1 N_1 S_1 $^{1-}$

Compound Name: Ammonium bis(N,N'-ethylene-bis(salicylideniminato)-nickel(ii)) isothiocyanate

Space Group:	Pbca	***Cell:***	***a***	16.873(1)	***b***	19.005(1)	***c***	20.058(1)
Space Group No.:	61	***(Å,°)***	α	90.00	β	90.00	γ	90.00

R-Factor (%): 3.94 ***Temperature(K):*** 291 ***Density(g/cm^3):*** 1.500

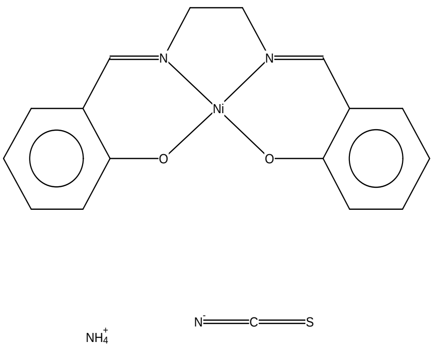

Parameters

Fragment 1

**DIST1 (D)**	3.614
**DIST2 (D)**	1.839
**DIST3 (D)**	1.842
**DIST4 (D)**	1.839
**DIST5 (D)**	1.848
**ANG1 (A)**	12.301

Fragment 2

**DIST1 (D)**	3.614
**DIST2 (D)**	1.842
**DIST3 (D)**	1.839
**DIST4 (D)**	1.848
**DIST5 (D)**	1.850
**ANG1 (A)**	7.974

NORFOR

Reference: Ali Hossein Kianfar, M.Dostani, W.A.K.Mahmood (2015) *Polyhedron* ,**85,**488

Formula: C_{16} H_{14} N_2 Ni_1 O_2,C_8 H_2 Cl_2 N_2 O_2

Compound Name: (2,2'-(ethane-1,2-diylbis((nitrilo)methylylidene))diphenolato)-nickel(ii) 4,5-dichloro-3,6-dihydroxyphthalonitrile

Space Group:	P-1	***Cell:***	***a***	8.566(0)	***b***	11.156(0)	***c***	13.913(1)
Space Group No.:	2	***(Å,°)***	α	111.97(0)	β	103.45(0)	γ	92.79(0)

R-Factor (%): 4.21 ***Temperature(K):*** 100 ***Density(g/cm^3):*** 1.552

Parameters

Fragment 1

**DIST1 (D)**	3.636
**DIST2 (D)**	1.853
**DIST3 (D)**	1.852
**DIST4 (D)**	1.843
**DIST5 (D)**	1.874
**ANG1 (A)**	6.179

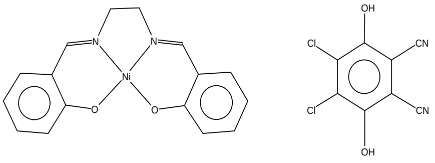

SAENNI

Reference: L.M.Shkol'nikova, E.M.Yumal', E.A.Shugam, V.A.Voblikova (1970) *Zh.Strukt.Khim.(Russ.)(J.Struct.Chem.)* ,**11,**886

Formula: C_{16} H_{14} N_2 Ni_1 O_2

Compound Name: (N,N'-bis(Salicylal)-ethylenedi-iminato)-nickel(ii)

Synonym: (2,2'-(ethane-1,2-diylbis((azanylylidene)methylylidene))bis(phenolato))-nickel(ii)

Space Group:	Pbca	***Cell:***	***a***	13.818(8)	***b***	26.132(9)	***c***	7.483(3)
Space Group No.:	61	***(Å,°)***	α	90.00	β	90.00	γ	90.00

R-Factor (%): 12.70 ***Temperature(K):*** 295 ***Density(g/cm^3):*** 1.597

Parameters

Fragment 1

**DIST1 (D)**	3.203
**DIST2 (D)**	1.867
**DIST3 (D)**	1.849
**DIST4 (D)**	1.831
**DIST5 (D)**	1.827
**ANG1 (A)**	7.173

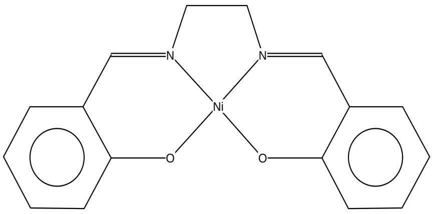

SAENNI01

Reference: A.G.Manfredotti, C.Guastini (1983) *Acta Crystallogr.,Sect.C:Cryst.Struct.Commun.* ,**39,**863

Formula: C_{16} H_{14} N_2 Ni_1 O_2

Compound Name: (N,N'-Ethylene-bis(salicylideneiminato))-nickel(ii)

Space Group:	Pbca	***Cell:***	***a***	13.831(3)	***b***	26.155(5)	***c***	7.482(2)
Space Group No.:	61	***(Å,°)***	α	90.00	β	90.00	γ	90.00

R-Factor (%): 4.20 ***Temperature(K):*** 295 ***Density(g/cm^3):*** 1.595

Parameters

Fragment 1

**DIST1 (D)**	3.201
**DIST2 (D)**	1.853
**DIST3 (D)**	1.843
**DIST4 (D)**	1.850
**DIST5 (D)**	1.854
**ANG1 (A)**	7.003

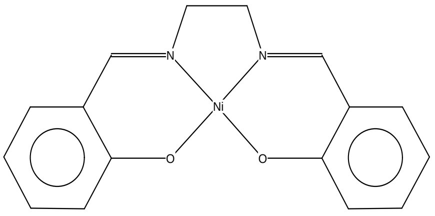

Search: search7 (Wed Jan 30 14:32:42 2019): Hits 13-16

SAENNI02

Reference: E.F.DiMauro, M.C.Kozlowski (2002) *Organometallics* ,**21,** 1454

Formula: $C_{16} H_{14} N_2 Ni_1 O_2$

Compound Name: (N,N'-Ethylene-bis(salicylideneaminato-N,O))-nickel(ii)

Space Group:	Pbca	***Cell:***	***a***	7.429(0)	***b***	13.740(0)	***c***	26.069(0)
Space Group No.:	61	***(Å,°)***	α	90.00	β	90.00	γ	90.00
R-Factor (%):	6.29	***Temperature(K):***	200	***Density(g/cm^3):***	1.623			

Parameters

Fragment 1	
**DIST1 (D)**	3.180
**DIST2 (D)**	1.850
**DIST3 (D)**	1.850
**DIST4 (D)**	1.850
**DIST5 (D)**	1.851
**ANG1 (A)**	6.946

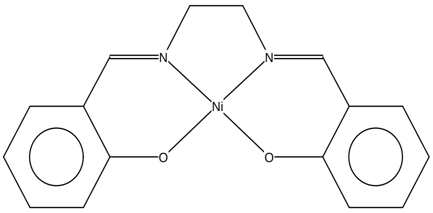

SAENNI03

Reference: M.Kondo, K.Nabari, T.Horiba, Y.Irie, Md K.Kabir, R.P.Sarker, E.Shimizu, Y.Shimizu, Y.Fuwa (2003) *Inorg.Chem.Commun.* ,**6,**154

Formula: $C_{16} H_{14} N_2 Ni_1 O_2$

Compound Name: (N,N'-Ethylene-bis(salicylideneiminato))-nickel(ii)

Space Group:	Pbca	***Cell:***	***a***	7.484(3)	***b***	13.822(6)	***c***	26.150(10)
Space Group No.:	61	***(Å,°)***	α	90.00	β	90.00	γ	90.00
R-Factor (%):	4.76	***Temperature(K):***	293	***Density(g/cm^3):***	1.596			

Parameters

Fragment 1	
**DIST1 (D)**	3.201
**DIST2 (D)**	1.846
**DIST3 (D)**	1.853
**DIST4 (D)**	1.852
**DIST5 (D)**	1.851
**ANG1 (A)**	6.759

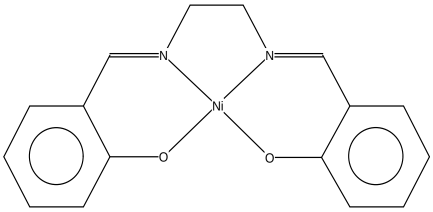

SAENNI05

Reference: M.A.Siegler, M.Lutz (2009) *Cryst.Growth Des.* ,**9,**1194

Formula: $C_{16} H_{14} N_2 Ni_1 O_2$

Compound Name: (N,N'-Ethylene-bis(salicylideneiminato))-nickel(ii)

Space Group:	Pcab	***Cell:***	***a***	13.684(0)	***b***	7.377(0)	***c***	25.968(0)
Space Group No.:	61	***(Å,°)***	α	90.00	β	90.00	γ	90.00
R-Factor (%):	1.86	***Temperature(K):***	100	***Density(g/cm^3):***	1.647			

Parameters

Fragment 1	
**DIST1 (D)**	3.160
**DIST2 (D)**	1.852
**DIST3 (D)**	1.849
**DIST4 (D)**	1.849
**DIST5 (D)**	1.854
**ANG1 (A)**	6.913

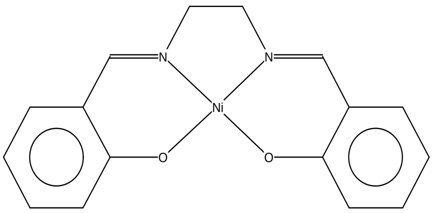

SAENNI06

Reference: Y.Ding, F.Wang, Z.J.Ku, L.S.Wang, Q.R.Wang (2009) *Koord.Khim.(Russ.)(Coord.Chem.)* ,**35,**366

Formula: $C_{16} H_{14} N_2 Ni_1 O_2$

Compound Name: (Ethylene-1,2-bis(salicylideneaminato))-nickel(ii)

Space Group:	Pbca	***Cell:***	***a***	7.472(0)	***b***	13.805(0)	***c***	26.098(1)
Space Group No.:	61	***(Å,°)***	α	90.00	β	90.00	γ	90.00
R-Factor (%):	2.38	***Temperature(K):***	295	***Density(g/cm^3):***	1.604			

Parameters

Fragment 1	
**DIST1 (D)**	3.196
**DIST2 (D)**	1.845
**DIST3 (D)**	1.850
**DIST4 (D)**	1.852
**DIST5 (D)**	1.845
**ANG1 (A)**	6.699

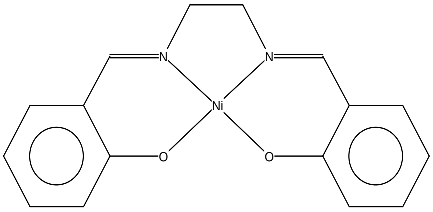

Search: search7 (Wed Jan 30 14:32:42 2019): Hits 17-20

SAENNI07

Reference: J.G.Malecki (2012)
CSD Communication(Private Communication) ,

Formula: $C_{16} H_{14} N_2 Ni_1 O_2$

Compound Name: (N,N'-Ethylenebis(salicylideneiminato))-nickel(ii)

Space Group:	Pbca	***Cell:***	***a***	7.474(1)	***b***	13.808(3)	***c***	26.143(5)
Space Group No.:	61	***(Å,°)***	α	90.00	β	90.00	γ	90.00

R-Factor (%): 3.95 ***Temperature(K):*** 295 ***Density(g/cm³):*** 1.600

Parameters

Fragment 1

**DIST1 (D)**	3.198
**DIST2 (D)**	1.852
**DIST3 (D)**	1.845
**DIST4 (D)**	1.852
**DIST5 (D)**	1.850
**ANG1 (A)**	6.596

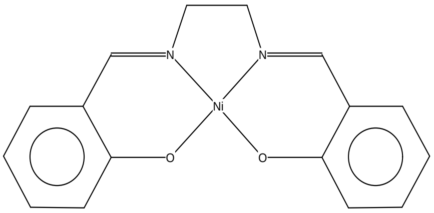

TUNREB

Reference: R.L.De, M.Mandal, L.Roy, J.Mukherjee, R.K.De (2014)
J.Indian Chem.Soc. ,**91,**2165

Formula: $C_{32} H_{32} N_4 O_6 Zn_3\ ^{2+}$,2($C_{16} H_{14} N_2 Ni_1 O_2$),2($Cl_1 O_4\ ^{1-}$),0.5($H_2 O_1$)

Compound Name: bis(μ_2-2,2'-(Ethane-1,2-diylbis(nitrilomethylylidene))diphenolato)-diaqua-tri-zinc (2,2'-(ethane-1,2-diylbis(nitrilomethylylidene))diphenolato)-nickel diperchlorate hemihydrate

Space Group:	C2/c	***Cell:***	***a***	28.527(2)	***b***	10.175(0)	***c***	24.161(2)
Space Group No.:	15	***(Å,°)***	α	90.00	β	111.89(0)	γ	90.00

R-Factor (%): 6.48 ***Temperature(K):*** 293 ***Density(g/cm³):*** 1.656

Parameters

Fragment 1

**DIST1 (D)**	3.281
**DIST2 (D)**	1.837
**DIST3 (D)**	1.836
**DIST4 (D)**	1.853
**DIST5 (D)**	1.858
**ANG1 (A)**	1.744

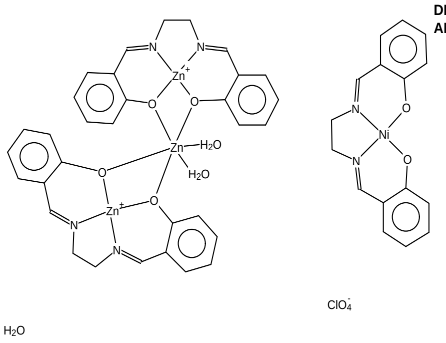

UMIBAT

Reference: M.Lutz (2003) *Acta Crystallogr.,Sect.E:Struct.Rep.Online ,*
59,m950

Formula: $C_{16} H_{14} N_2 Ni_1 O_2$,$C_3 H_7 N_1 O_1$

Compound Name: (N,N'-Ethylene-bis(salicylideneiminato-N,O))-nickel(ii) dimethylformamide solvate

Space Group:	P21/c	***Cell:***	***a***	13.387(0)	***b***	6.669(0)	***c***	22.733(0)
Space Group No.:	14	***(Å,°)***	α	90.00	β	118.04(0)	γ	90.00

R-Factor (%): 2.70 ***Temperature(K):*** 150 ***Density(g/cm³):*** 1.476

Parameters

Fragment 1

**DIST1 (D)**	3.390
**DIST2 (D)**	1.848
**DIST3 (D)**	1.845
**DIST4 (D)**	1.845
**DIST5 (D)**	1.850
**ANG1 (A)**	7.132

Fragment 2

**DIST1 (D)**	3.551
**DIST2 (D)**	1.848
**DIST3 (D)**	1.845
**DIST4 (D)**	1.845
**DIST5 (D)**	1.850
**ANG1 (A)**	7.132

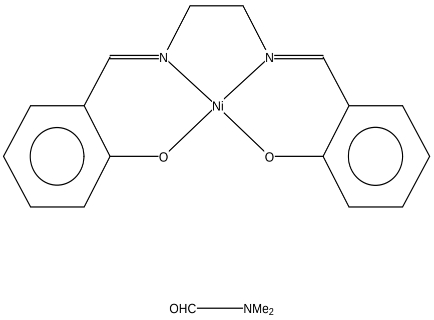

VUYJAB

Reference: G.Assey, Y.Gultneh, R.J.Butcher (2010)
Acta Crystallogr.,Sect.E:Struct.Rep.Online ,**66,**m654

Formula: $C_2 H_{10} N_2\ ^{2+}$,4($C_{16} H_{14} N_2 Ni_1 O_2$),2($Cl_1 O_4\ ^{1-}$),2($C_3 H_7 N_1 O_1$)

Compound Name: Ethylenediammonium tetrakis((2,2'-[ethane-1,2-diylbis(nitrilomethylidyne)]diphenolato)-nickel(ii)) diperchlorate N,N-dimethylformamide solvate

Space Group:	P-1	***Cell:***	***a***	15.021(1)	***b***	15.049(1)	***c***	18.271(0)
Space Group No.:	2	***(Å,°)***	α	85.99(0)	β	86.51(0)	γ	62.96(0)

R-Factor (%): 3.89 ***Temperature(K):*** 200 ***Density(g/cm³):*** 1.546

Parameters

Fragment 1

**DIST1 (D)**	3.454
**DIST2 (D)**	1.844
**DIST3 (D)**	1.850
**DIST4 (D)**	1.838
**DIST5 (D)**	1.852
**ANG1 (A)**	11.970

Fragment 2

**DIST1 (D)**	3.454
**DIST2 (D)**	1.848
**DIST3 (D)**	1.837
**DIST4 (D)**	1.849
**DIST5 (D)**	1.837
**ANG1 (A)**	2.806

Fragment 3

**DIST1 (D)**	3.644
**DIST2 (D)**	1.846
**DIST3 (D)**	1.845
**DIST4 (D)**	1.841
**DIST5 (D)**	1.856
**ANG1 (A)**	10.723

Fragment 4

**DIST1 (D)**	3.644
**DIST2 (D)**	1.850
**DIST3 (D)**	1.842
**DIST4 (D)**	1.856
**DIST5 (D)**	1.845
**ANG1 (A)**	13.120

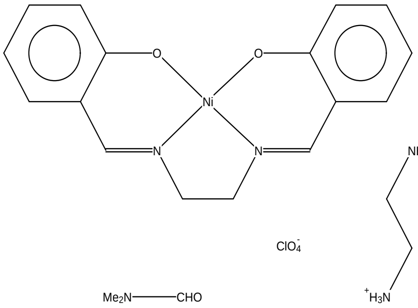

Supplement: Supplementary file 3 [file e-75-00328-sup3.pdf]
