# Supplementary material for: Crystal structure and Hirshfeld surfaces analysis of the nickel(II) complex of the Shiff base ligand 6,6′-{(1E,1′E)-[ethane-1,2-diylbis(aza­nylyl­idene)]bis­(methanylyl­idene)}bis­[2-(tri­fluoro­meth­oxy)phenol]
Source: Acta Crystallogr E Crystallogr Commun. 2019 Feb 8;75(Pt 3):328–31. doi: 10.1107/S2056989019001919 (PMC6399694; doi:10.1107/S2056989019001919)
Supplement: Supplementary file 4 [file e-75-00328-sup4.pdf]

Search Overview

Search:	search8
Date/Time done:	Wed Jan 30 14:36:05 2019
Database(s):	CSD version 5.40 (November 2018)
Restriction Info:	No refcode restrictions applied
Filters:	None
Percentage Completed:	100%
Number of Hits:	11

Single query used. Search found structures that:

match

Query 1

Query 1

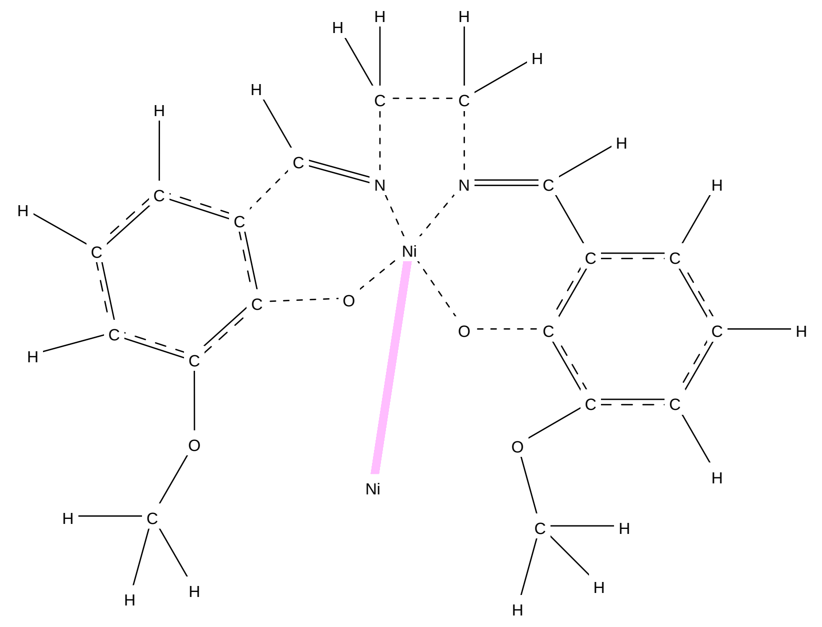

Search: search8 (Wed Jan 30 14:36:05 2019): Hits 1-4

AXUTOE

Reference: S.Hazra, R.Meyrelles, A.J.Charmier, P.Rijo, M.Fatima C.Guedes da Silva, A.J.L.Pombeiro (2016) *Dalton Trans.* ,**45,** 17929

Formula: $2(C_{18}H_{18}N_2Ni_1O_4),C_2H_{10}N_2{}^{2+},Cl_6Sn_1{}^{2-}$

Compound Name: ethane-1,2-diaminium bis((2,2'-(ethane-1,2-diylbis(iminomethylidene)) bis(6-methoxyphenolato))-nickel(ii)) hexachloro-tin(iv)

Space Group:	P-1	***Cell:***	***a*** 10.489(1)	***b*** 10.924(1)	***c*** 11.138(1)		
Space Group No.:	2	***(Å,°)***	α 90.22(0)	β 117.44(0)	γ 99.73(0)		
R-Factor (%):	5.09	***Temperature(K):***	298	***Density(g/cm³):***	1.738		

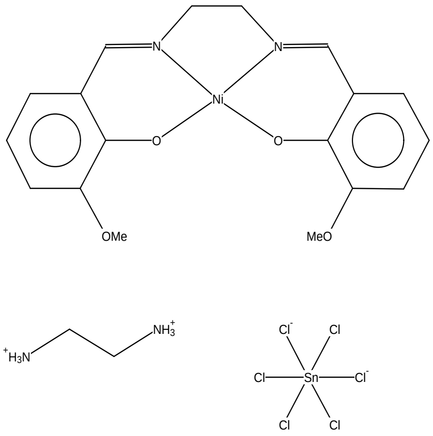

Parameters

Fragment 1

**DIST1 (D)**	3.757
**DIST2 (D)**	1.851
**DIST3 (D)**	1.842
**DIST4 (D)**	1.861
**DIST5 (D)**	1.871
**ANG1 (A)**	19.077

IQEFAM

Reference: A.Cucos, A.Ursu, A.M.Madalan, C.Duhayon, J.-P.Sutter, M.Andruh (2011) *CrystEngComm* ,**13,**3756

Formula: $C_{18}H_{18}N_2Ni_1O_4,0.5(H_4N_1{}^{1+}),0.5(Cl_1O_4{}^{1-})$

Compound Name: hemiammonium hemiperchlorate (N,N'-bis(3-methoxysalicylidene) ethylenediamine)-nickel(ii)

Space Group:	P-1	***Cell:***	***a*** 11.528(1)	***b*** 12.787(1)	***c*** 15.627(1)
Space Group No.:	2	***(Å,°)***	α 65.75(0)	β 74.11(0)	γ 65.06(0)
R-Factor (%):	5.85	***Temperature(K):***	293	***Density(g/cm³):***	1.559

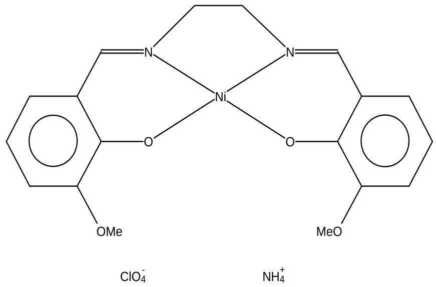

Parameters

Fragment 1

**DIST1 (D)**	3.365
**DIST2 (D)**	1.860
**DIST3 (D)**	1.845
**DIST4 (D)**	1.866
**DIST5 (D)**	1.843
**ANG1 (A)**	3.382

Fragment 2

**DIST1 (D)**	3.430
**DIST2 (D)**	1.841
**DIST3 (D)**	1.850
**DIST4 (D)**	1.843
**DIST5 (D)**	1.850
**ANG1 (A)**	12.510

IQEFEQ

Reference: A.Cucos, A.Ursu, A.M.Madalan, C.Duhayon, J.-P.Sutter, M.Andruh (2011) *CrystEngComm* ,**13,**3756

Formula: $C_{18}H_{18}N_2Ni_1O_4,0.5(H_4N_1{}^{1+}),0.5(F_6P_1{}^{1-})$

Compound Name: hemiammonium hemikis(hexafluorophosphate) (N,N'-bis(3-methoxysalicylidene)ethylenediamine)-nickel(ii)

Space Group:	P-1	***Cell:***	***a*** 11.680(1)	***b*** 12.873(1)	***c*** 15.748(1)
Space Group No.:	2	***(Å,°)***	α 66.15(0)	β 73.26(0)	γ 64.35(0)
R-Factor (%):	6.03	***Temperature(K):***	293	***Density(g/cm³):***	1.603

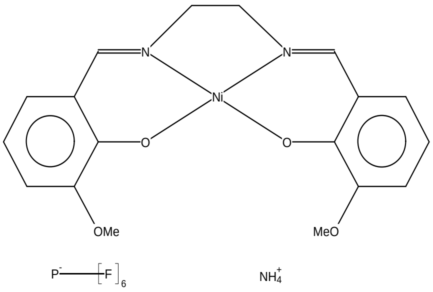

Parameters

Fragment 1

**DIST1 (D)**	3.425
**DIST2 (D)**	1.844
**DIST3 (D)**	1.862
**DIST4 (D)**	1.857
**DIST5 (D)**	1.858
**ANG1 (A)**	3.669

Fragment 2

**DIST1 (D)**	3.430
**DIST2 (D)**	1.842
**DIST3 (D)**	1.846
**DIST4 (D)**	1.855
**DIST5 (D)**	1.842
**ANG1 (A)**	11.935

IQEFOA

Reference: A.Cucos, A.Ursu, A.M.Madalan, C.Duhayon, J.-P.Sutter, M.Andruh (2011) *CrystEngComm* ,**13,**3756

Formula: $2(C_{18}H_{18}N_2Ni_1O_4),1.5(H_4N_1{}^{1+}),1.5(Cl_1O_4{}^{1-})$

Compound Name: sesquiammonium sesquiperchlorate bis((N,N'-bis(3-methoxysalicylidene) ethylenediamine)-nickel(ii))

Space Group:	P-1	***Cell:***	***a*** 14.844(1)	***b*** 15.699(1)	***c*** 17.358(1)
Space Group No.:	2	***(Å,°)***	α 97.49(0)	β 97.83(0)	γ 91.79(0)
R-Factor (%):	5.63	***Temperature(K):***	293	***Density(g/cm³):***	1.584

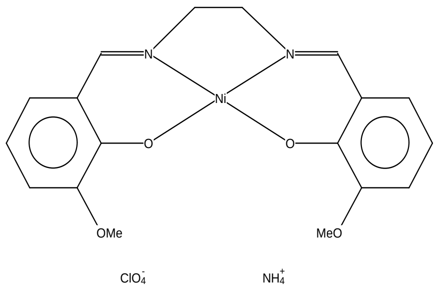

Parameters

Fragment 1

**DIST1 (D)**	3.349
**DIST2 (D)**	1.860
**DIST3 (D)**	1.839
**DIST4 (D)**	1.843
**DIST5 (D)**	1.852
**ANG1 (A)**	5.351

Fragment 2

**DIST1 (D)**	3.378
**DIST2 (D)**	1.834
**DIST3 (D)**	1.847
**DIST4 (D)**	1.858
**DIST5 (D)**	1.849
**ANG1 (A)**	8.298

Fragment 3

**DIST1 (D)**	3.879
**DIST2 (D)**	1.849
**DIST3 (D)**	1.832
**DIST4 (D)**	1.842
**DIST5 (D)**	1.855
**ANG1 (A)**	3.678

Fragment 4

**DIST1 (D)**	3.879
**DIST2 (D)**	1.860
**DIST3 (D)**	1.839
**DIST4 (D)**	1.843
**DIST5 (D)**	1.852
**ANG1 (A)**	5.351

Search: search8 (Wed Jan 30 14:36:05 2019): Hits 5-8

TEJGIZ

Reference: Yu-Ye Yu (2006) *Acta Crystallogr.,Sect.E:Struct.Rep.Online* ,**62,**m948

Formula: $C_{18} H_{18} N_2 Ni_1 O_4, C_1 H_1 Cl_3$

Compound Name: 6,6'-Dimethoxy-2,2'-(ethane-1,2-diylbis(nitrilomethylidyne)diphenolato)-nickel(ii) chloroform solvate

**Space Group:**	P21/c	**Cell:**	**a**	11.381(2)	**b**	13.842(3)	**c**	13.268(3)
**Space Group No.:**	14	**(Å,°)**	α	90.00	β	91.43(3)	γ	90.00

R-Factor (%): 4.09 **Temperature(K):** 296 **Density(g/cm^3):** 1.603

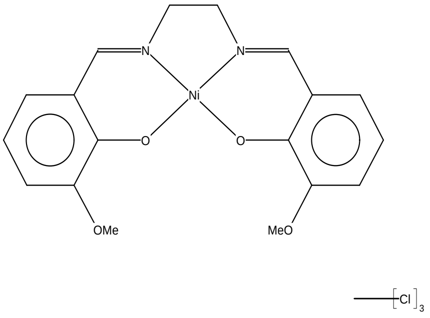

Parameters

Fragment 1

**DIST1 (D)**	3.448
**DIST2 (D)**	1.846
**DIST3 (D)**	1.843
**DIST4 (D)**	1.839
**DIST5 (D)**	1.846
**ANG1 (A)**	3.398

ULAMIE

Reference: K.Ayikoe, R.J.Butcher, Y.Gultneh (2011) *Acta Crystallogr.,Sect.E:Struct.Rep.Online* ,**67,**m328

Formula: $C_{18} H_{18} N_2 Ni_1 O_4, C_3 H_7 N_1 O_1$

Compound Name: (6,6'-Dimethoxy-2,2'-[ethane-1,2-diylbis(nitrilomethanylidene)]diphenolato)-nickel(ii) dimethylformamide solvate

**Space Group:**	P21/c	**Cell:**	**a**	6.860(0)	**b**	15.343(0)	**c**	18.907(0)
**Space Group No.:**	14	**(Å,°)**	α	90.00	β	91.68(0)	γ	90.00

R-Factor (%): 4.14 **Temperature(K):** 110 **Density(g/cm^3):** 1.530

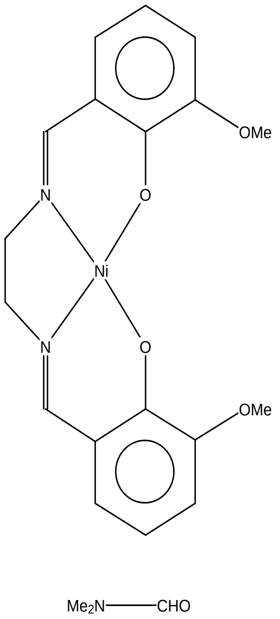

Parameters

Fragment 1

**DIST1 (D)**	3.534
**DIST2 (D)**	1.850
**DIST3 (D)**	1.850
**DIST4 (D)**	1.861
**DIST5 (D)**	1.859
**ANG1 (A)**	3.875

Fragment 2

**DIST1 (D)**	3.617
**DIST2 (D)**	1.850
**DIST3 (D)**	1.850
**DIST4 (D)**	1.861
**DIST5 (D)**	1.859
**ANG1 (A)**	3.875

WAVYOH

Reference: D.Cunningham, J.F.Gallagher, T.Higgins, P.McArdle, J.McGinley, M.O'Gara (1993) *J.Chem.Soc.,Dalton Trans.* ,2183

Formula: $C_{18} H_{18} N_2 Ni_1 O_4, C_2 H_8 Cl_2 O_1 Sn_1, C_1 H_1 Cl_3$

Compound Name: (N,N'-bis(3-Methoxysalicylidene)ethylene-1,2-diamine-O,O',N,N')-nickel(ii) aqua-dichloro-dimethyl-tin(iv) chloroform solvate

**Space Group:**	P-1	**Cell:**	**a**	9.986(2)	**b**	9.972(2)	**c**	15.246(3)
**Space Group No.:**	2	**(Å,°)**	α	88.03(3)	β	75.33(7)	γ	87.90(6)

R-Factor (%): 5.00 **Temperature(K):** 295 **Density(g/cm^3):** 1.680

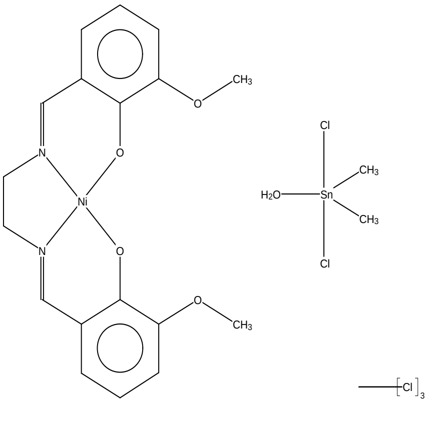

Parameters

Fragment 1

**DIST1 (D)**	3.401
**DIST2 (D)**	1.828
**DIST3 (D)**	1.862
**DIST4 (D)**	1.849
**DIST5 (D)**	1.856
**ANG1 (A)**	2.278

XEMJOQ

Reference: D.Zhang, Z.Zhao (2011) *Koord.Khim.(Russ.)(Coord.Chem.)* ,**37,**934

Formula: $4(C_{18} H_{18} N_2 Ni_1 O_4), 3(Cl_1 O_4\ ^{1-}), 3(K_1\ ^{1+})$

Compound Name: tri-potassium tetrakis((2,2'-(ethane-1,2-diylbis((imino)methyl))bis(6-methoxyphenolato))-nickel(ii)) triperchlorate

**Space Group:**	P-1	**Cell:**	**a**	14.666(1)	**b**	15.769(1)	**c**	17.287(1)
**Space Group No.:**	2	**(Å,°)**	α	97.87(0)	β	98.15(0)	γ	91.73(0)

R-Factor (%): 5.64 **Temperature(K):** 295 **Density(g/cm^3):** 1.659

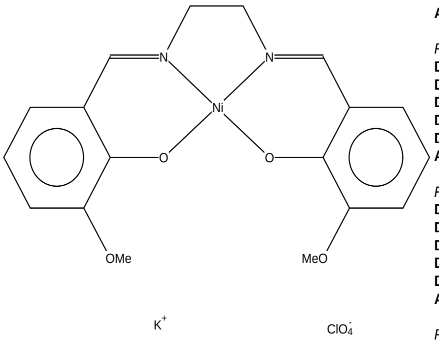

Parameters

Fragment 1

**DIST1 (D)**	3.367
**DIST2 (D)**	1.848
**DIST3 (D)**	1.850
**DIST4 (D)**	1.843
**DIST5 (D)**	1.834
**ANG1 (A)**	4.334

Fragment 2

**DIST1 (D)**	3.429
**DIST2 (D)**	1.852
**DIST3 (D)**	1.843
**DIST4 (D)**	1.848
**DIST5 (D)**	1.848
**ANG1 (A)**	8.959

Fragment 3

**DIST1 (D)**	3.834
**DIST2 (D)**	1.842
**DIST3 (D)**	1.838
**DIST4 (D)**	1.833
**DIST5 (D)**	1.843
**ANG1 (A)**	3.569

Fragment 4

**DIST1 (D)**	3.834
**DIST2 (D)**	1.848
**DIST3 (D)**	1.850
**DIST4 (D)**	1.843
**DIST5 (D)**	1.834
**ANG1 (A)**	4.334
