# Supplementary material for: Crystal structure and Hirshfeld surfaces analysis of the nickel(II) complex of the Shiff base ligand 6,6′-{(1E,1′E)-[ethane-1,2-diylbis(aza­nylyl­idene)]bis­(methanylyl­idene)}bis­[2-(tri­fluoro­meth­oxy)phenol]
Source: Acta Crystallogr E Crystallogr Commun. 2019 Feb 8;75(Pt 3):328–31. doi: 10.1107/S2056989019001919 (PMC6399694; doi:10.1107/S2056989019001919)
Supplement: Supplementary file 5 [file e-75-00328-sup5.pdf]

Search Overview

Search: search9

Date/Time done: Wed Jan 30 14:37:24 2019

Database(s): CSD version 5.40 (November 2018)

Restriction Info: No refcode restrictions applied

Filters: None

Percentage Completed: 100%

Number of Hits: 26

Single query used. Search found structures that:

match

Query 1

Query 1

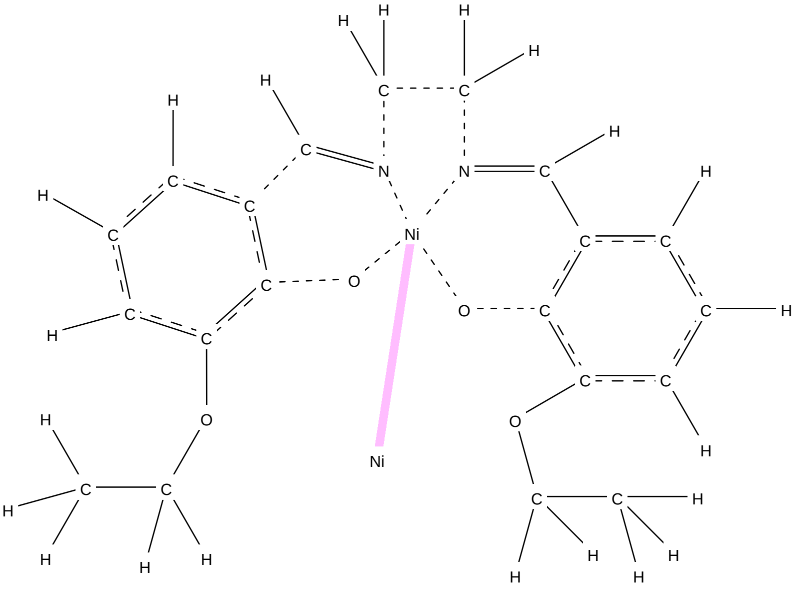

Search: search9 (Wed Jan 30 14:37:24 2019): Hits 1-4

AXUTUK

Reference: S.Hazra, R.Meyrelles, A.J.Charmier, P.Rijo, M.Fatima C.Guedes da Silva, A.J.L.Pombeiro (2016) *Dalton Trans.* ,**45,** 17929

Formula: 2(C_{20} H_{22} N_2 Ni_1 O_4),C_2 H_{10} N_2 $^{2+}$,Cl_6 Sn_1 $^{2-}$,2(C_1 H_4 O_1)

Compound Name: ethane-1,2-diaminium bis((2,2'-(ethane-1,2-diylbis(iminomethylidene)) bis(6-ethoxyphenolato))-nickel(ii)) hexachloro-tin(iv) methanol solvate

Space Group:	P-1	***Cell:***	***a*** 10.317(0)	***b*** 11.751(0)	***c*** 12.822(0)		
Space Group No.:	2	***(Å,°)***	α 114.36(0)	β 108.04(0)	γ 96.32(0)		

R-Factor (%): 2.67 ***Temperature(K):*** 150 ***Density(g/cm³):*** 1.646

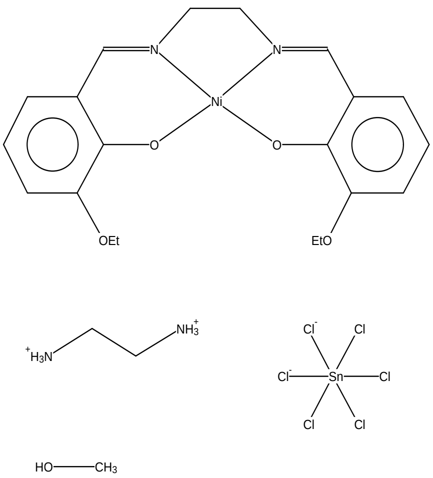

Parameters

Fragment 1

**DIST1 (D)**	3.840
**DIST2 (D)**	1.856
**DIST3 (D)**	1.852
**DIST4 (D)**	1.867
**DIST5 (D)**	1.874
**ANG1 (A)**	5.114

ECAYEO

Reference: S.Sarkar, S.Mohanta (2011) *RSC Advances* ,**1,**640

Formula: C_{20} H_{26} Li_1 N_2 Ni_1 O_6 $^{1+}$,2(C_{20} H_{22} N_2 Ni_1 O_4),Cl_1 O_4 $^{1-}$

Compound Name: (μ_2-2,2'-(ethane-1,2-diylbis((nitrilo)methylylidene))-bis(6-ethoxyphenolato))-diaqua-lithium-nickel(ii) bis((2,2'-(ethane-1,2-diylbis((nitrilo)methylylidene))-bis(6-ethoxyphenolato))-nickel(ii)) perchlorate

Space Group:	P-1	***Cell:***	***a*** 11.861(1)	***b*** 15.811(2)	***c*** 17.483(2)
Space Group No.:	2	***(Å,°)***	α 84.90(0)	β 73.44(0)	γ 80.68(0)

R-Factor (%): 5.24 ***Temperature(K):*** 296 ***Density(g/cm³):*** 1.481

Parameters

Fragment 1

**DIST1 (D)**	3.324
**DIST2 (D)**	1.834
**DIST3 (D)**	1.840
**DIST4 (D)**	1.840
**DIST5 (D)**	1.839
**ANG1 (A)**	4.334

Fragment 2

**DIST1 (D)**	3.373
**DIST2 (D)**	1.845
**DIST3 (D)**	1.835
**DIST4 (D)**	1.841
**DIST5 (D)**	1.851
**ANG1 (A)**	6.760

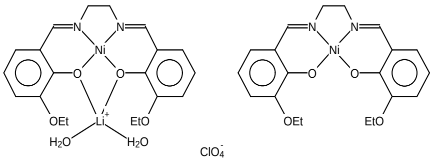

ECAYIS

Reference: S.Sarkar, S.Mohanta (2011) *RSC Advances* ,**1,**640

Formula: 2(C_{20} H_{22} N_2 Ni_1 O_4),H_4 N_1 $^{1+}$,F_6 P_1 $^{1-}$

Compound Name: ammonium bis((2,2'-(ethane-1,2-diylbis((nitrilo)methylylidene))-bis(6-ethoxyphenolato))-nickel(ii)) hexafluorophosphate

Space Group:	P21/n	***Cell:***	***a*** 15.934(3)	***b*** 14.920(2)	***c*** 18.255(3)
Space Group No.:	14	***(Å,°)***	α 90.00	β 92.11(0)	γ 90.00

R-Factor (%): 4.36 ***Temperature(K):*** 296 ***Density(g/cm³):*** 1.515

Parameters

Fragment 1

**DIST1 (D)**	3.434
**DIST2 (D)**	1.845
**DIST3 (D)**	1.848
**DIST4 (D)**	1.840
**DIST5 (D)**	1.844
**ANG1 (A)**	3.596

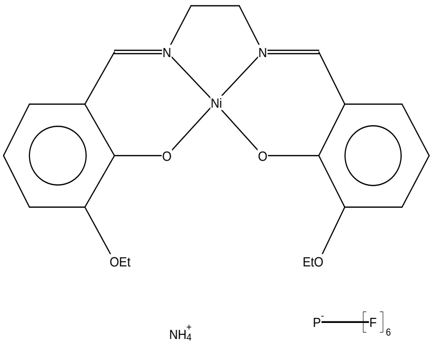

ECAZIT

Reference: S.Sarkar, S.Mohanta (2011) *RSC Advances* ,**1,**640

Formula: C_{20} H_{28} Mg_1 N_2 Ni_1 O_7 $^{2+}$,2(C_{20} H_{22} N_2 Ni_1 O_4),2(Cl_1 O_4 $^{1-}$)

Compound Name: (μ_2-2,2'-(ethane-1,2-diylbis((nitrilo)methylylidene))-bis(6-ethoxyphenolato))-triaqua-magnesium-nickel(ii) bis((2,2'-(ethane-1,2-diylbis((nitrilo)methylylidene))-bis(6-ethoxyphenolato))-nickel(ii)) diperchlorate

Space Group:	C2/c	***Cell:***	***a*** 15.442(1)	***b*** 22.455(1)	***c*** 18.848(1)
Space Group No.:	15	***(Å,°)***	α 90.00	β 94.56(0)	γ 90.00

R-Factor (%): 5.12 ***Temperature(K):*** 296 ***Density(g/cm³):*** 1.546

Parameters

Fragment 1

**DIST1 (D)**	3.450
**DIST2 (D)**	1.844
**DIST3 (D)**	1.838
**DIST4 (D)**	1.854
**DIST5 (D)**	1.841
**ANG1 (A)**	11.197

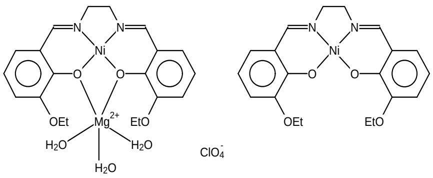

Search: search9 (Wed Jan 30 14:37:24 2019): Hits 5-8

GECFEB

Reference: S.Sarkar, M.Fleck, S.Mohanta (2012) *J.Mol.Struct.* ,**1021,** 174

Formula: $C_2 H_{10} N_2{}^{2+}$,2($C_{20} H_{22} N_2 Ni_1 O_4$),2($N_1 O_3{}^{1-}$)

Compound Name: Ethane-1,2-diammonium (2,2'-(ethane-1,2-diylbis(nitrilomethylylidene))-bis(6-ethoxyphenolato))-nickel(ii) dinitrate

Space Group:	P-1	***Cell:***	***a*** 9.290(2)	***b*** 11.419(2)	***c*** 11.909(2)
Space Group No.:	2	***(Å,°)***	α 111.09(0)	β 97.86(0)	γ 93.02(0)

R-Factor (%): 3.85 ***Temperature(K):*** 296 ***Density(g/cm^3):*** 1.449

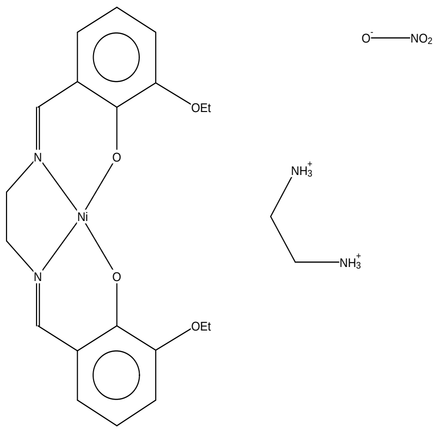

Parameters

Fragment 1

**DIST1 (D)**	3.915
**DIST2 (D)**	1.864
**DIST3 (D)**	1.863
**DIST4 (D)**	1.867
**DIST5 (D)**	1.870
**ANG1 (A)**	19.547

GECFIF

Reference: S.Sarkar, M.Fleck, S.Mohanta (2012) *J.Mol.Struct.* ,**1021,** 174

Formula: $C_3 H_{12} N_2{}^{2+}$,2($C_{20} H_{22} N_2 Ni_1 O_4$),2($N_1 O_3{}^{1-}$)

Compound Name: Propane-1,2-diammonium (2,2'-(ethane-1,2-diylbis(nitrilomethylylidene))-bis(6-ethoxyphenolato))-nickel(ii) dinitrate

Space Group:	P1	***Cell:***	***a*** 9.241(0)	***b*** 11.528(0)	***c*** 12.066(0)
Space Group No.:	1	***(Å,°)***	α 114.46(0)	β 98.45(0)	γ 91.47(0)

R-Factor (%): 5.31 ***Temperature(K):*** 296 ***Density(g/cm^3):*** 1.479

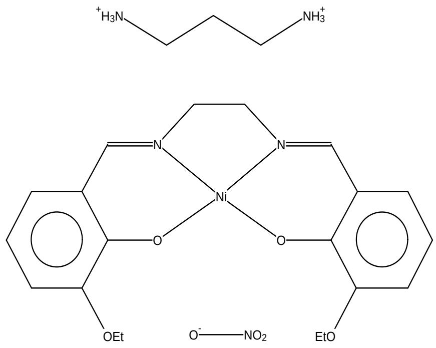

Parameters

Fragment 1

**DIST1 (D)**	3.849
**DIST2 (D)**	1.860
**DIST3 (D)**	1.838
**DIST4 (D)**	1.849
**DIST5 (D)**	1.835
**ANG1 (A)**	19.342

Fragment 2

**DIST1 (D)**	3.849
**DIST2 (D)**	1.867
**DIST3 (D)**	1.843
**DIST4 (D)**	1.871
**DIST5 (D)**	1.873
**ANG1 (A)**	20.876

TUMMUL

Reference: S.Ghosh, L.Mandal, S.Mohanta (2015) *Polyhedron* ,**97,**1

Formula: $C_{20} H_{22} N_2 Ni_1 O_4$,0.5($C_4 H_4 O_4$),$H_2 O_1$

Compound Name: (2,2'-(ethane-1,2-diylbis(nitrilomethylylidene))bis(6-ethoxyphenolato))-nickel(ii) hemikis(but-2-enedioate) monohydrate

Synonym: (2,2'-(ethane-1,2-diylbis(nitrilomethylylidene))bis(6-ethoxyphenolato))-nickel(ii) hemikis(fumaric acid) monohydrate

Space Group:	P21/c	***Cell:***	***a*** 9.633(0)	***b*** 15.398(1)	***c*** 14.908(1)
Space Group No.:	14	***(Å,°)***	α 90.00	β 94.12(0)	γ 90.00

R-Factor (%): 3.58 ***Temperature(K):*** 296 ***Density(g/cm^3):*** 1.473

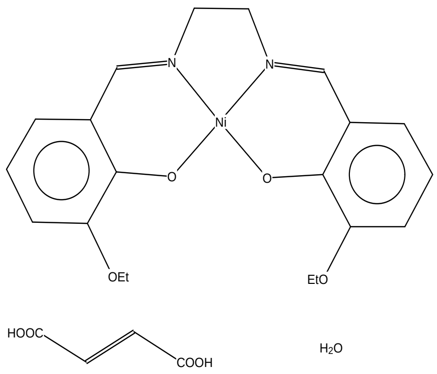

Parameters

Fragment 1

**DIST1 (D)**	3.422
**DIST2 (D)**	1.843
**DIST3 (D)**	1.843
**DIST4 (D)**	1.845
**DIST5 (D)**	1.846
**ANG1 (A)**	7.343

TUMNEW

Reference: S.Ghosh, L.Mandal, S.Mohanta (2015) *Polyhedron* ,**97,**1

Formula: $C_{20} H_{22} N_2 Ni_1 O_4$,0.5($C_4 H_6 O_4$),$H_2 O_1$

Compound Name: (2,2'-(ethane-1,2-diylbis((nitrilo)methylylidene))bis(6-ethoxyphenolato))-nickel(ii) hemikis(succinic acid) monohydrate

Space Group:	P21/c	***Cell:***	***a*** 9.495(4)	***b*** 15.361(7)	***c*** 15.067(7)
Space Group No.:	14	***(Å,°)***	α 90.00	β 93.93(0)	γ 90.00

R-Factor (%): 5.21 ***Temperature(K):*** 296 ***Density(g/cm^3):*** 1.485

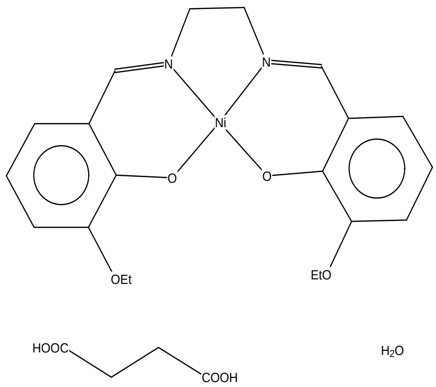

Parameters

Fragment 1

**DIST1 (D)**	3.391
**DIST2 (D)**	1.827
**DIST3 (D)**	1.827
**DIST4 (D)**	1.829
**DIST5 (D)**	1.836
**ANG1 (A)**	7.935

Search: search9 (Wed Jan 30 14:37:24 2019): Hits 9-12

TUMNIA

Reference: S.Ghosh, L.Mandal, S.Mohanta (2015) *Polyhedron* ,**97,**1

Formula: $C_{20}\,H_{22}\,N_2\,Ni_1\,O_4$,0.5($C_8\,H_6\,O_4$),$H_2\,O_1$

Compound Name: (2,2'-(ethane-1,2-diylbis((nitrilo)methylylidene))bis(6-ethoxyphenolato))-nickel(ii) hemikis(terephthalic acid) monohydrate

Space Group:	P-1	***Cell:***	***a*** 7.055(0)	***b*** 13.094(1)	***c*** 13.140(1)		
Space Group No.:	2	***(Å,°)***	α 90.71(0)	β 91.94(0)	γ 102.98(0)		
R-Factor (%):	3.84	***Temperature(K):***	296	***Density(g/cm³):***	1.445		

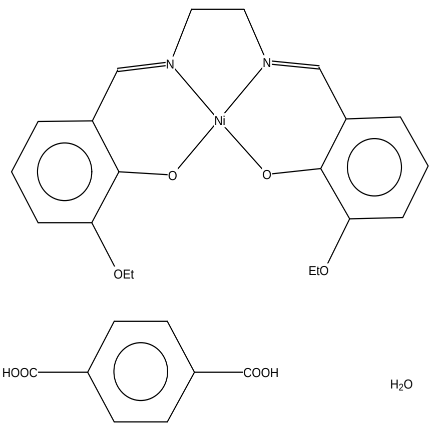

Parameters

Fragment 1	
**DIST1 (D)**	3.311
**DIST2 (D)**	1.844
**DIST3 (D)**	1.853
**DIST4 (D)**	1.855
**DIST5 (D)**	1.853
**ANG1 (A)**	3.664

Fragment 2	
**DIST1 (D)**	3.969
**DIST2 (D)**	1.844
**DIST3 (D)**	1.853
**DIST4 (D)**	1.855
**DIST5 (D)**	1.853
**ANG1 (A)**	3.664

TUMNUM

Reference: S.Ghosh, L.Mandal, S.Mohanta (2015) *Polyhedron* ,**97,**1

Formula: $C_{20}\,H_{22}\,N_2\,Ni_1\,O_4$,$C_9\,H_6\,O_6$,$C_1\,H_4\,O_1$,$H_2\,O_1$

Compound Name: (2,2'-(ethane-1,2-diylbis(nitrilomethylylidene))bis(6-ethoxyphenolato))-nickel(ii) benzene-1,3,5-tricarboxylic acid methanol solvate monohydrate

Space Group:	P-1	***Cell:***	***a*** 7.949(1)	***b*** 11.857(1)	***c*** 17.187(2)
Space Group No.:	2	***(Å,°)***	α 92.95(0)	β 95.72(0)	γ 100.30(0)
R-Factor (%):	4.75	***Temperature(K):***	296	***Density(g/cm³):***	1.414

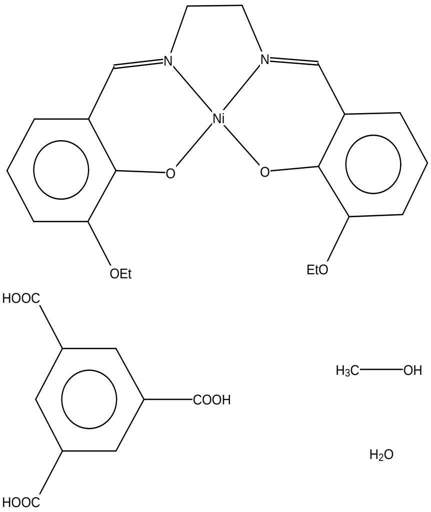

Parameters

Fragment 1	
**DIST1 (D)**	3.410
**DIST2 (D)**	1.860
**DIST3 (D)**	1.847
**DIST4 (D)**	1.851
**DIST5 (D)**	1.849
**ANG1 (A)**	10.683

VOHSIV

Reference: Hai Xie (2008) *Acta Crystallogr.,Sect.E:Struct.Rep.Online* , **64,**m1638

Formula: $C_{20}\,H_{22}\,N_2\,Ni_1\,O_4$,$H_2\,O_1$

Compound Name: (2,2'-(1,2-Ethanediylbis((nitrilo-κN)methylylidene))-bis(6-ethoxyphenolato))-nickel(ii) monohydrate

Space Group:	Pbcn	***Cell:***	***a*** 12.840(0)	***b*** 19.613(1)	***c*** 7.585(0)
Space Group No.:	60	***(Å,°)***	α 90.00	β 90.00	γ 90.00
R-Factor (%):	2.71	***Temperature(K):***	273	***Density(g/cm³):***	1.499

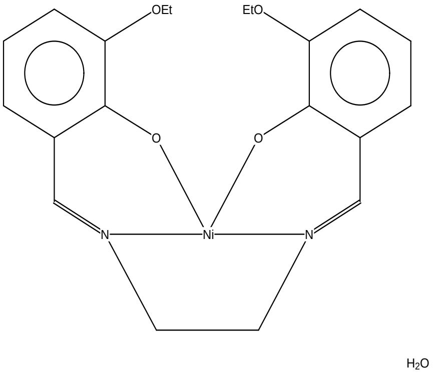

Parameters

Fragment 1	
**DIST1 (D)**	3.904
**DIST2 (D)**	1.846
**DIST3 (D)**	1.846
**DIST4 (D)**	1.865
**DIST5 (D)**	1.865
**ANG1 (A)**	19.868

VOHSIV01

Reference: S.Sarkar, M.Nayak, M.Fleck, S.Dutta, U.Florke, R.Koner, S.Mohanta (2010) *Eur.J.Inorg.Chem.* ,735

Formula: $C_{20}\,H_{22}\,N_2\,Ni_1\,O_4$,$H_2\,O_1$

Compound Name: (2,2'-(1,2-Ethanediylbis((nitrilo-N)methylylidene))-bis(6-ethoxyphenolato))-nickel(ii) monohydrate

Space Group:	Pbcn	***Cell:***	***a*** 12.778(1)	***b*** 19.344(3)	***c*** 7.510(1)
Space Group No.:	60	***(Å,°)***	α 90.00	β 90.00	γ 90.00
R-Factor (%):	2.34	***Temperature(K):***	120	***Density(g/cm³):***	1.543

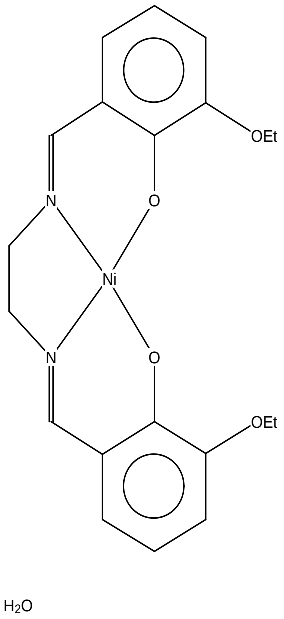

Parameters

Fragment 1	
**DIST1 (D)**	3.862
**DIST2 (D)**	1.850
**DIST3 (D)**	1.850
**DIST4 (D)**	1.864
**DIST5 (D)**	1.864
**ANG1 (A)**	21.117

Search: search9 (Wed Jan 30 14:37:24 2019): Hits 13-16

XUWCUO

Reference: S.Sarkar, M.Nayak, M.Fleck, S.Dutta, U.Florke, R.Koner, S.Mohanta (2010) *Eur.J.Inorg.Chem.* ,735

Formula: $2(C_{20}\,H_{22}\,N_2\,Ni_1\,O_4),C_{20}\,H_{28}\,N_2\,Ni_2\,O_7\,^{2+},2(Cl_1\,O_4\,^{1-})$

Compound Name: bis(N,N'-Ethylene-bis(3-ethoxysalicylaldimine)-nickel(ii)) (μ_2-N,N'-ethylene-bis(3-ethoxysalicylaldimine))-triaqua-di-nickel(ii) diperchlorate

Space Group:	C2/c	***Cell:***	***a*** 15.488(0)	***b*** 22.400(0)	***c*** 18.700(0)		
Space Group No.:	15	***(Å,°)***	α 90.00	β 94.29(0)	γ 90.00		
R-Factor (%):	8.32	***Temperature(K):***	293	***Density(g/cm^3):***	1.592		

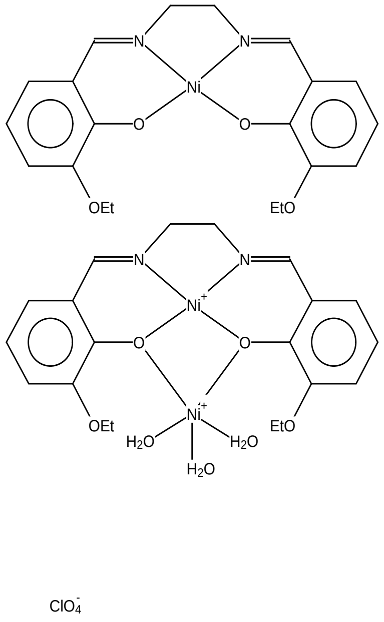

Parameters

Fragment 1

**DIST1 (D)**	3.470
**DIST2 (D)**	1.844
**DIST3 (D)**	1.842
**DIST4 (D)**	1.845
**DIST5 (D)**	1.843
**ANG1 (A)**	11.313

XUWCUO01

Reference: S.Sarkar, M.Nayak, M.Fleck, S.Dutta, U.Florke, R.Koner, S.Mohanta (2010) *Eur.J.Inorg.Chem.* ,735

Formula: $2(C_{20}\,H_{22}\,N_2\,Ni_1\,O_4),C_{20}\,H_{28}\,N_2\,Ni_2\,O_7\,^{2+},2(Cl_1\,O_4\,^{1-})$

Compound Name: bis(N,N'-Ethylene-bis(3-ethoxysalicylaldimine)-nickel(ii)) (μ_2-N,N'-ethylene-bis(3-ethoxysalicylaldimine))-triaqua-di-nickel(ii) diperchlorate

Space Group:	Cc	***Cell:***	***a*** 15.488(1)	***b*** 22.400(1)	***c*** 18.700(1)
Space Group No.:	9	***(Å,°)***	α 90.00	β 94.29(0)	γ 90.00
R-Factor (%):	7.64	***Temperature(K):***	293	***Density(g/cm^3):***	1.592

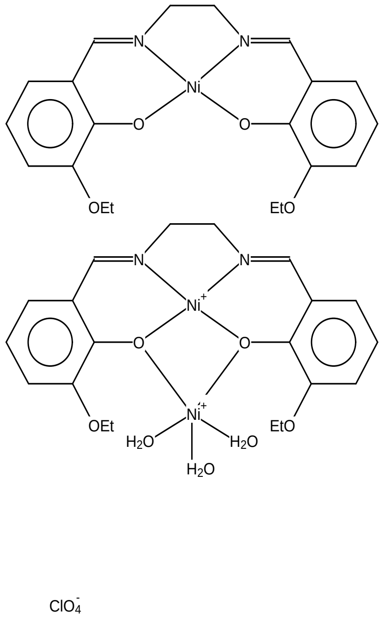

Parameters

Fragment 1

**DIST1 (D)**	3.470
**DIST2 (D)**	1.752
**DIST3 (D)**	1.843
**DIST4 (D)**	1.837
**DIST5 (D)**	1.842
**ANG1 (A)**	11.530

Fragment 2

**DIST1 (D)**	3.470
**DIST2 (D)**	1.859
**DIST3 (D)**	1.934
**DIST4 (D)**	1.860
**DIST5 (D)**	1.841
**ANG1 (A)**	12.049

XUWDAV

Reference: S.Sarkar, M.Nayak, M.Fleck, S.Dutta, U.Florke, R.Koner, S.Mohanta (2010) *Eur.J.Inorg.Chem.* ,735

Formula: $2(C_{20}\,H_{22}\,N_2\,Ni_1\,O_4),C_{20}\,H_{28}\,Co_1\,N_2\,Ni_1\,O_7\,^{2+},2(Cl_1\,O_4\,^{1-})$

Compound Name: bis(N,N'-Ethylene-bis(3-ethoxysalicylaldimine)-nickel(ii)) (μ_2-N,N'-ethylene-bis(3-ethoxysalicylaldimine))-triaqua-nickel(ii)-cobalt(ii) diperchlorate

Space Group:	C2/c	***Cell:***	***a*** 15.458(1)	***b*** 22.452(1)	***c*** 18.800(1)
Space Group No.:	15	***(Å,°)***	α 90.00	β 94.60(0)	γ 90.00
R-Factor (%):	4.64	***Temperature(K):***	293	***Density(g/cm^3):***	1.584

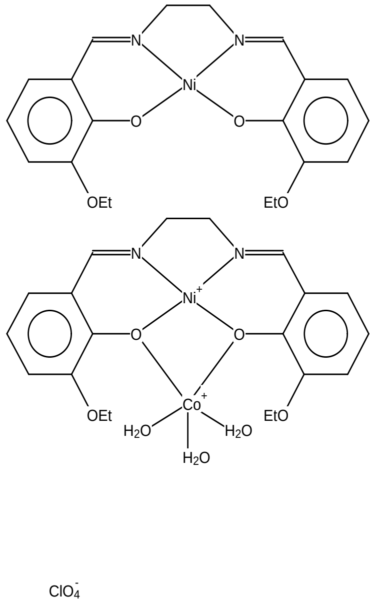

Parameters

Fragment 1

**DIST1 (D)**	3.458
**DIST2 (D)**	1.852
**DIST3 (D)**	1.846
**DIST4 (D)**	1.854
**DIST5 (D)**	1.844
**ANG1 (A)**	11.828

XUWDAV01

Reference: S.Sarkar, M.Nayak, M.Fleck, S.Dutta, U.Florke, R.Koner, S.Mohanta (2010) *Eur.J.Inorg.Chem.* ,735

Formula: $2(C_{20}\,H_{22}\,N_2\,Ni_1\,O_4),C_{20}\,H_{28}\,Co_1\,N_2\,Ni_1\,O_7\,^{2+},2(Cl_1\,O_4\,^{1-})$

Compound Name: bis(N,N'-Ethylene-bis(3-ethoxysalicylaldimine)-nickel(ii)) (μ_2-N,N'-ethylene-bis(3-ethoxysalicylaldimine))-triaqua-nickel(ii)-cobalt(ii) diperchlorate

Space Group:	Cc	***Cell:***	***a*** 15.458(1)	***b*** 22.452(1)	***c*** 18.800(1)
Space Group No.:	9	***(Å,°)***	α 90.00	β 94.60(0)	γ 90.00
R-Factor (%):	4.10	***Temperature(K):***	293	***Density(g/cm^3):***	1.584

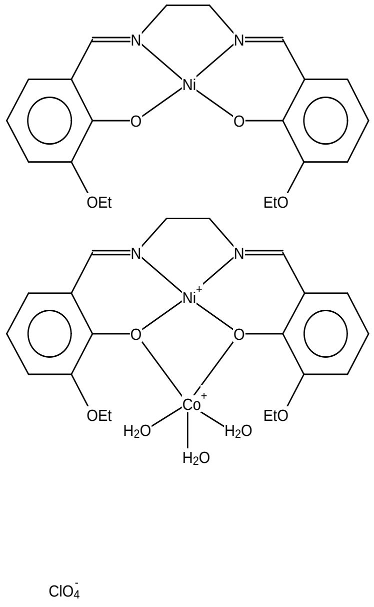

Parameters

Fragment 1

**DIST1 (D)**	3.458
**DIST2 (D)**	1.803
**DIST3 (D)**	1.836
**DIST4 (D)**	1.820
**DIST5 (D)**	1.867
**ANG1 (A)**	11.727

Fragment 2

**DIST1 (D)**	3.458
**DIST2 (D)**	1.865
**DIST3 (D)**	1.889
**DIST4 (D)**	1.847
**DIST5 (D)**	1.865
**ANG1 (A)**	12.172

Search: search9 (Wed Jan 30 14:37:24 2019): Hits 17-20

XUWDEZ

Reference: S.Sarkar, M.Nayak, M.Fleck, S.Dutta, U.Florke, R.Koner, S.Mohanta (2010) *Eur.J.Inorg.Chem.* ,735

Formula: $2(C_{20} H_{22} N_2 Ni_1 O_4),C_{20} H_{28} Fe_1 N_2 Ni_1 O_7\ {}^{2+},2(Cl_1 O_4\ {}^{1-})$

Compound Name: bis(N,N'-Ethylene-bis(3-ethoxysalicylaldimine)-nickel(ii)) (μ_2-N,N'-ethylene-bis(3-ethoxysalicylaldimine))-triaqua-nickel(ii)-iron(ii) diperchlorate

Space Group: C2/c ***Cell:*** ***a*** 15.439(0) ***b*** 22.414(0) ***c*** 18.779(0)
Space Group No.: 15 ***(Å,°)*** α 90.00 β 94.32(0) γ 90.00

R-Factor (%): 5.82 ***Temperature(K):*** 293 ***Density(g/cm³):*** 1.587

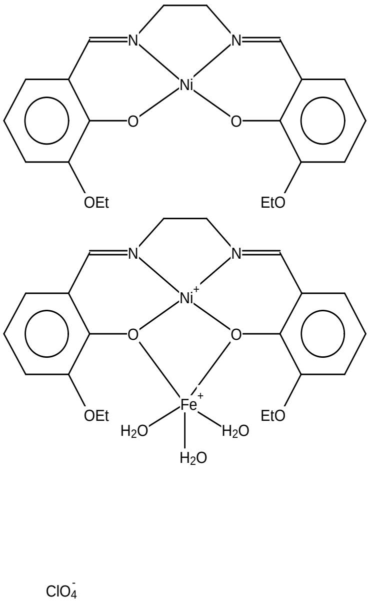

Parameters

Fragment 1

**DIST1 (D)**	3.445
**DIST2 (D)**	1.850
**DIST3 (D)**	1.842
**DIST4 (D)**	1.855
**DIST5 (D)**	1.843
**ANG1 (A)**	11.799

XUWDEZ01

Reference: S.Sarkar, M.Nayak, M.Fleck, S.Dutta, U.Florke, R.Koner, S.Mohanta (2010) *Eur.J.Inorg.Chem.* ,735

Formula: $2(C_{20} H_{22} N_2 Ni_1 O_4),C_{20} H_{28} Fe_1 N_2 Ni_1 O_7\ {}^{2+},2(Cl_1 O_4\ {}^{1-})$

Compound Name: bis(N,N'-Ethylene-bis(3-ethoxysalicylaldimine)-nickel(ii)) (μ_2-N,N'-ethylene-bis(3-ethoxysalicylaldimine))-triaqua-nickel(ii)-iron(ii) diperchlorate

Space Group: Cc ***Cell:*** ***a*** 15.439(0) ***b*** 22.414(0) ***c*** 18.779(0)
Space Group No.: 9 ***(Å,°)*** α 90.00 β 94.32(0) γ 90.00

R-Factor (%): 5.55 ***Temperature(K):*** 293 ***Density(g/cm³):*** 1.587

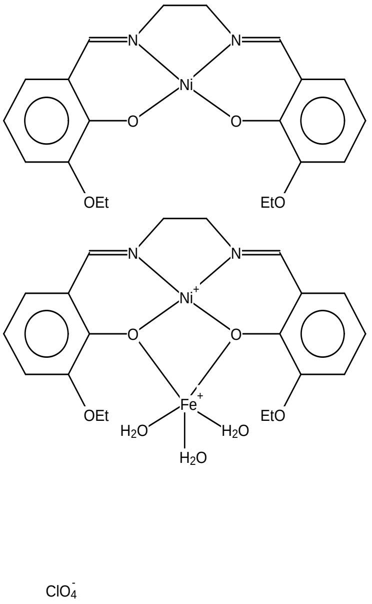

Parameters

Fragment 1

**DIST1 (D)**	3.446
**DIST2 (D)**	1.815
**DIST3 (D)**	1.819
**DIST4 (D)**	1.820
**DIST5 (D)**	1.848
**ANG1 (A)**	12.003

Fragment 2

**DIST1 (D)**	3.446
**DIST2 (D)**	1.880
**DIST3 (D)**	1.880
**DIST4 (D)**	1.861
**DIST5 (D)**	1.866
**ANG1 (A)**	11.638

XUWDID

Reference: S.Sarkar, M.Nayak, M.Fleck, S.Dutta, U.Florke, R.Koner, S.Mohanta (2010) *Eur.J.Inorg.Chem.* ,735

Formula: $2(C_{20} H_{22} N_2 Ni_1 O_4),C_{20} H_{28} Mn_1 N_2 Ni_1 O_7\ {}^{2+},2(Cl_1 O_4\ {}^{1-})$

Compound Name: bis(N,N'-Ethylene-bis(3-ethoxysalicylaldimine)-nickel(ii)) (μ_2-N,N'-ethylene-bis(3-ethoxysalicylaldimine))-triaqua-nickel(ii)-manganese(ii) diperchlorate

Space Group: C2/c ***Cell:*** ***a*** 15.437(0) ***b*** 22.444(0) ***c*** 18.855(0)
Space Group No.: 15 ***(Å,°)*** α 90.00 β 94.68(0) γ 90.00

R-Factor (%): 4.27 ***Temperature(K):*** 293 ***Density(g/cm³):*** 1.578

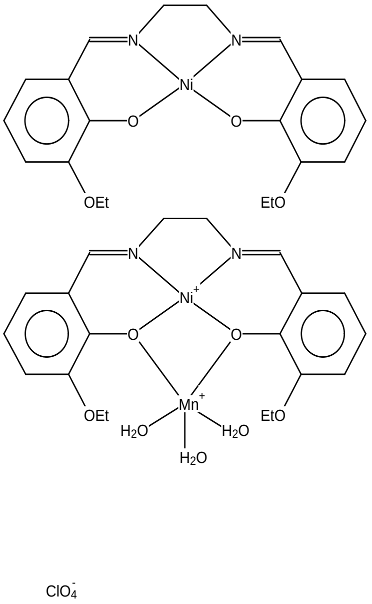

Parameters

Fragment 1

**DIST1 (D)**	3.451
**DIST2 (D)**	1.849
**DIST3 (D)**	1.846
**DIST4 (D)**	1.851
**DIST5 (D)**	1.845
**ANG1 (A)**	11.920

XUWDID01

Reference: S.Sarkar, M.Nayak, M.Fleck, S.Dutta, U.Florke, R.Koner, S.Mohanta (2010) *Eur.J.Inorg.Chem.* ,735

Formula: $2(C_{20} H_{22} N_2 Ni_1 O_4),C_{20} H_{28} Mn_1 N_2 Ni_1 O_7\ {}^{2+},2(Cl_1 O_4\ {}^{1-})$

Compound Name: bis(N,N'-Ethylene-bis(3-ethoxysalicylaldimine)-nickel(ii)) (μ_2-N,N'-ethylene-bis(3-ethoxysalicylaldimine))-triaqua-nickel(ii)-manganese(ii) diperchlorate

Space Group: Cc ***Cell:*** ***a*** 15.437(0) ***b*** 22.444(0) ***c*** 18.855(0)
Space Group No.: 9 ***(Å,°)*** α 90.00 β 94.68(0) γ 90.00

R-Factor (%): 3.97 ***Temperature(K):*** 293 ***Density(g/cm³):*** 1.578

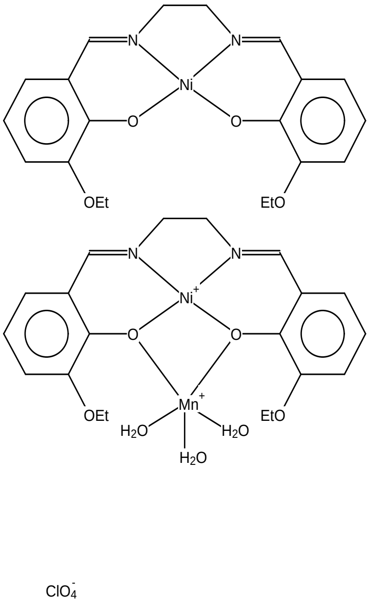

Parameters

Fragment 1

**DIST1 (D)**	3.451
**DIST2 (D)**	1.839
**DIST3 (D)**	1.825
**DIST4 (D)**	1.843
**DIST5 (D)**	1.847
**ANG1 (A)**	12.509

Fragment 2

**DIST1 (D)**	3.451
**DIST2 (D)**	1.869
**DIST3 (D)**	1.855
**DIST4 (D)**	1.844
**DIST5 (D)**	1.857
**ANG1 (A)**	11.323

Search: search9 (Wed Jan 30 14:37:24 2019): Hits 21-22

XUWDOJ

Reference: S.Sarkar, M.Nayak, M.Fleck, S.Dutta, U.Florke, R.Koner, S.Mohanta (2010) *Eur.J.Inorg.Chem.* ,735

Formula: $2(C_{20} H_{22} N_2 Ni_1 O_4),C_{20} H_{28} Cu_1 N_2 Ni_1 O_7{}^{2+},2(Cl_1 O_4{}^{1-})$

Compound Name: bis(N,N'-Ethylene-bis(3-ethoxysalicylaldimine)-nickel(ii)) (μ_2-N,N'-ethylene-bis(3-ethoxysalicylaldimine))-triaqua-nickel(ii)-copper(ii) diperchlorate

Space Group:	Cc	***Cell:***	***a*** 15.387(2)	***b*** 22.127(2)	***c*** 18.424(2)		
Space Group No.:	9	***(Å,°)***	α 90.00	β 93.79(0)	γ 90.00		
R-Factor (%):	5.14	***Temperature(K):***	100	***Density(g/cm^3):***	1.651		

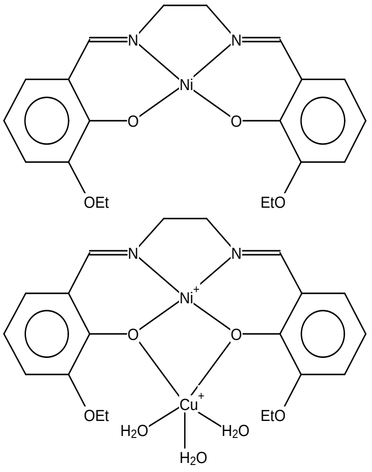

ClO_4^-

Parameters

Fragment 1

**DIST1 (D)**	3.384
**DIST2 (D)**	1.836
**DIST3 (D)**	1.865
**DIST4 (D)**	1.881
**DIST5 (D)**	1.837
**ANG1 (A)**	13.738

Fragment 2

**DIST1 (D)**	3.384
**DIST2 (D)**	1.841
**DIST3 (D)**	1.869
**DIST4 (D)**	1.854
**DIST5 (D)**	1.829
**ANG1 (A)**	12.004

XUWDOJ01

Reference: S.Sarkar, M.Nayak, M.Fleck, S.Dutta, U.Florke, R.Koner, S.Mohanta (2010) *Eur.J.Inorg.Chem.* ,735

Formula: $2(C_{20} H_{22} N_2 Ni_1 O_4),C_{20} H_{28} Cu_1 N_2 Ni_1 O_7{}^{2+},2(Cl_1 O_4{}^{1-})$

Compound Name: bis(N,N'-Ethylene-bis(3-ethoxysalicylaldimine)-nickel(ii)) (μ_2-N,N'-ethylene-bis(3-ethoxysalicylaldimine))-triaqua-nickel(ii)-copper(ii) diperchlorate

Space Group:	C2/c	***Cell:***	***a*** 15.449(1)	***b*** 22.242(1)	***c*** 18.423(1)
Space Group No.:	15	***(Å,°)***	α 90.00	β 95.15(0)	γ 90.00
R-Factor (%):	5.85	***Temperature(K):***	120	***Density(g/cm^3):***	1.639

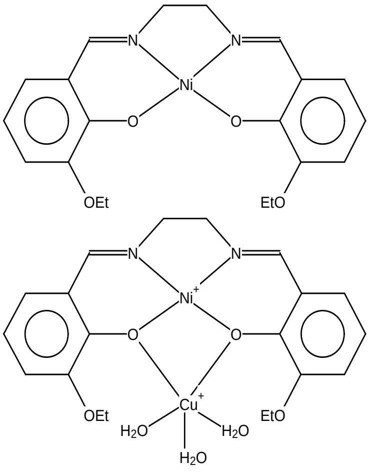

ClO_4^-

Parameters

Fragment 1

**DIST1 (D)**	3.511
**DIST2 (D)**	1.874
**DIST3 (D)**	1.892
**DIST4 (D)**	1.858
**DIST5 (D)**	1.869
**ANG1 (A)**	16.757
